# Standardising management of consent withdrawal and other clinical trial participation changes: The UKCRC Registered Clinical Trials Unit Network’s PeRSEVERE project

**DOI:** 10.1177/17407745251344524

**Published:** 2025-07-04

**Authors:** William J Cragg, Laura Clifton-Hadley, Jeremy Dearling, Susan J Dutton, Katie Gillies, Pollyanna Hardy, Daniel Hind, Søren Holm, Kerenza Hood, Anna Kearney, Rebecca Lewis, Sarah Markham, Lauren Moreau, Tra My Pham, Amanda Roberts, Sharon Ruddock, Mirjana Sirovica, Ratna Sohanpal, Puvan Tharmanathan, Rejina Verghis

**Affiliations:** 1 Clinical Trials Research Unit, Leeds Institute of Clinical Trials Research, University of Leeds, Leeds, UK; 2 Cancer Research UK and University College London Cancer Trials Centre, University College London, London, UK; 3 Patient Contributor, UK; 4 Oxford Clinical Trials Research Unit and Centre for Statistics in Medicine, University of Oxford, Oxford, UK; 5 Health Services Research Unit, University of Aberdeen, Aberdeen, UK; 6 National Perinatal Epidemiology Unit and Clinical Trials Unit, Nuffield Department of Population Health, University of Oxford, Oxford, UK; 7 School of Healthcare, University of Leeds, Leeds, UK; 8 The University of Manchester, Manchester, UK; 9 University of Oslo, Oslo, Norway; 10 Centre for Trials Research, Cardiff University, Cardiff, UK; 11 Department of Health Data Science, University of Liverpool, Liverpool, UK; 12 Clinical Trials and Statistics Unit, The Institute of Cancer Research, London, UK; 13 King’s College London, London, UK; 14 Medical Research Council Clinical Trials Unit, University College London, London, UK; 15 Liverpool Clinical Trials Centre, University of Liverpool, Liverpool, UK; 16 Leicester Clinical Trials Unit, University of Leicester, Leicester, UK; 17 Centre for Primary Care, Wolfson Institute of Population Health, Queen Mary University of London, London, UK; 18 Department of Health Sciences, Faculty of Science, University of York, York, UK; 19 Wellcome-Wolfson Institute for Experimental Medicine, School of Medicine, Dentistry and Biomedical Sciences, Queen’s University Belfast, Belfast, UK

**Keywords:** Informed consent, withdrawal, retention, attrition, participation changes

## Abstract

**Background/Aims:**

Existing regulatory and ethical guidance does not address real-life complexities in how clinical trial participants’ level of participation may change. If these complexities are inappropriately managed, there may be negative consequences for trial participants and the integrity of trials they participate in. These concerns have been highlighted over many years, but there remains no single, comprehensive guidance for managing participation changes in ways that address real-life complexities while maximally promoting participant interests and trial integrity. Motivated by the lack of agreed standards, and observed variability in practice, representatives from academic clinical trials units and linked organisations in the United Kingdom initiated the PeRSEVERE project (PRincipleS for handling end-of-participation EVEnts in clinical trials REsearch) to agree on guiding principles and explore how these principles should be implemented.

**Methods:**

We developed the PeRSEVERE principles through discussion and debate within a large, multidisciplinary collaboration, including research professionals and public contributors. We took an inclusive approach to drafting the principles, incorporating new ideas if they were within project scope. Our draft principles were scrutinised through an international consultation survey which focussed on the principles’ clarity, feasibility, novelty and acceptability. Survey responses were analysed descriptively (for category questions) and using a combination of deductive and inductive analysis (for open questions). We used predefined rules to guide feedback handling. After finalising the principles, we developed accompanying implementation guidance from several sources.

**Results:**

In total, 280 people from 9 countries took part in the consultation survey. Feedback showed strong support for the principles with 96% of respondents agreeing with the principles’ key messages. Based on our predefined rules, it was not necessary to amend our draft principles, but comments were nonetheless used to enhance the final project outputs. Our 17 finalised principles comprise 7 fundamental, ‘overarching’ principles, 6 about trial design and setup, 2 covering data collection and monitoring, and 2 on trial analysis and reporting.

**Conclusion:**

We devised a comprehensive set of guiding principles, with detailed practical recommendations, to aid the management of clinical trial participation changes, building on existing ethical and regulatory texts. Our outputs reflect the contributions of a substantial number of individuals, including public contributors and research professionals with various specialisms. This lends weight to our recommendations, which have implications for everyone who designs, funds, conducts, oversees or participates in trials. We suggest our principles could lead to improved standards in clinical trials and better experiences for participants. We encourage others to build on our work to explore the application of these ideas in other settings and to generate empirical evidence to support best practice in this area.

## Introduction

It is well established in modern research ethics that clinical trial participants must retain control over their ongoing participation. This is mainly addressed through the ‘right to withdraw consent’ in relevant laws, policies and guidelines, which standardly says that participants may stop taking part at any time, without explanation or reprisal.^1–5^ Establishment of this right was essential following previous unethical practices and protects participants’ autonomy.^6,7^

However, the right does not address real-life complexities in the nature and extent of different ‘participation changes’ (used henceforth as a catch-all for any ways that participation can stop, reduce or change). The term ‘withdrawal’ itself, as used in the above-referenced sources, might imply that research participation has two states: continuation or discontinuation. In practice, many clinical trials comprise several participation elements, such as receiving intervention, completing questionnaires,^8^ attending follow-up visits or allowing data collection from routine clinic visits or central databases.^9^ It is often possible for participants to stop some elements while continuing others. For example, participants may no longer feel able to attend trial-specific clinic visits but may be happy to continue follow-up via another method (e.g. via routine healthcare appointments only). Existing policies also do not explain what should happen when contact is lost between researchers and participants, or when caregivers or those with a duty of care decide it is in participants’ interests for some aspects of participation to stop (e.g. receipt of trial intervention).

These limitations lead to potential challenges in practice. For example, they might lead to the assumption that *any* expressed participant wish to ‘withdraw’ from a trial must result in *all* aspects of their participation stopping. If this assumption leads to the loss of potentially available trial outcome data, it may unnecessarily impair trial integrity,^10–12^ devalue individual participants’ contributions (and those of other participants in the same trial) and could be considered research waste.^13^ It may also be unfair to participants who would be happy to continue participating in a more limited fashion or want to stay in touch and find out the results. Alternatively, if participants have the impression that they may only continue or stop all aspects of the trial, some may feel pressure to continue participating to avoid ‘letting the trial down’.^14^

This basic ‘right to withdraw’ has remained largely unchanged since the mid-20th century, despite important changes in the relationship between society and research during this time. Clinical research is now more commonly done collaboratively with patients and the public,^15^ improving the chances of producing ‘fruitful results for the good of society’.^16^ It is also accepted that research participation can benefit individuals, in a broad sense.^17–20^ By extension, if a participant is struggling to continue with all trial activities, continuing with *reduced* participation, instead of stopping all trial-associated activity and contact, could also benefit them. Failure to offer participants such reduced options when they should be possible may contradict the idea of participants having the right to withdraw consent ‘without penalty or loss of benefits’.^2^

Trialists have raised concerns about the management of participation change for many years, often in the context of preventing missing data^21–24^ but also highlighting deficiencies with researcher training,^25^ trial reporting^26^ and information given to potential participants.^27,28^ We are not aware of a single, comprehensive guidance resource for managing participation changes across trial design, conduct and analysis, that addresses real-life complexity and maximally promotes participants’ interests and trial integrity.

Motivated by this lack of agreed standards and the resulting variability observed in practice, representatives from academic clinical trials units within the UK Clinical Research Collaboration (UKCRC) Registered Clinical Trials Unit (CTU) Network^29^ formed a project called PeRSEVERE (PRincipleS for handling end-of-participation EVEnts in clinical trials REsearch). The project’s aims were (1) to develop principles, built on existing Good Clinical Practice (GCP) concepts, to guide how participation changes should be prepared for and handled across clinical trial design, setup, conduct, analysis and reporting and (2) to produce accompanying detailed guidance about implementing the principles. We report here the process we undertook to develop and seek feedback on the principles and guidance.

## Methods

**Figure 1.**
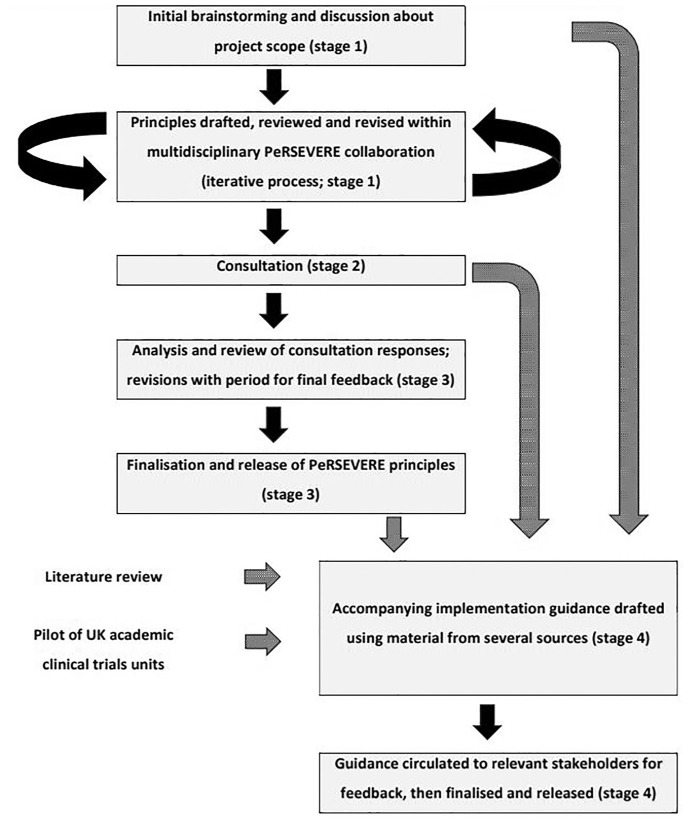
Overview of methods used to develop and consult on the PeRSEVERE principles.

Figure 1 summarises our methods. During all project stages, we aimed for an inclusive and deliberative approach, allowing incorporation of new ideas if they were agreed to be within the scope of our objectives (with the exact scope explored and defined through testing new suggestions), and allowing time to understand objections, find compromises and get buy-in on the final outputs. All feedback was recorded and categorised systematically, and text changes were documented and shared across the collaboration for transparency.

### Initial principle development (stage 1)

Ideas for principles were generated through a multidisciplinary meeting of representatives of 36/51 Network CTUs in October 2019, following preliminary work at the Clinical Trials Research Unit (CTRU), University of Leeds.

The principles were drafted and revised via an iterative process across seven topic-specific working groups, including statisticians, operational staff, CTU directors, research ethicists, academics and public contributors. Each working group’s leads formed a project steering group. Further detail about this initial work is available on the project website.^30^

### Consultation (stage 2)

We ran a cross-sectional consultation survey on our draft principles, to gather feedback from relevant interest-holders. A CROSS checklist^31^ has guided our reporting of this survey (see Supplementary Materials).

#### Survey design and dissemination

This survey was run online via the Online Surveys platform.^32^ Given the broad topic applicability, the survey was open to any interested individuals, with the only eligibility criterion excluding participation by PeRSEVERE steering group members. The survey and draft principles were provided in English.

We aimed for a balanced set of responses from a range of respondents, including people identifying as research professionals or not (e.g. public contributors), people working in industry and academia, based in different countries and with different personal characteristics. We sent the survey to research-relevant groups and individuals in the United Kingdom and internationally, with encouragement to share. Project team members sent the survey link via email, and others sharing on our behalf sent it through whichever route they considered appropriate. Given the nature of the exercise, we did not calculate a sample size but aimed for several hundred responses to give substantial feedback. Five Leeds CTRU staff unconnected to PeRSEVERE piloted the survey.

The survey protocol and question text are available in the Supplementary Materials. Respondents were asked to rate each draft principle on a 5-point Likert-type scale (‘Strongly Agree’, ‘Agree’, ‘Not sure’, ‘Disagree’, ‘Strongly Disagree’), in terms of its clarity, feasibility to implement, acceptability and novelty compared to current practice (using a conceptual framework adapted from the lead author’s prior work).^33^ Respondents were given space to suggest any important considerations unaddressed by our principles and to make any other, general comments.

Respondents could choose to provide feedback on just the principles’‘key messages’ and skip groups of principles to save time. After the main survey questions, we asked respondents for information about themselves, including personal characteristics (e.g. age and ethnicity) and information on their experience with research (based on questions and categories used in steering group members’ previous projects). During stage 1, we had devised suggested, improved terminology to support clearer communication about participation changes. Our survey asked respondents for views on these suggestions.

Individuals were required to indicate their informed consent to participate before contributing. The survey requested no identifiable data.

We obtained ethical approval from the University of Leeds School of Medicine Research Ethics Committee (reference MREC 20-060) before launching the survey.

#### Analysing survey responses

Analysis was primarily conducted by the project lead (W.J.C.) using Microsoft Excel. During analysis, any internal inconsistencies in the data were highlighted and suitable actions agreed upon within the steering group. Missing data were included in descriptive statistics, except where a quantitative response was missing but related free text responses unambiguously indicated an answer (e.g. ‘I completely agree’).

Per-principle quantitative questions were analysed descriptively by coding the responses 1–5, with 5 being ‘strongly agree’. We summarised responses by respondents’ self-identification as primarily research professional or not. Demographic questions were summarised descriptively. We conducted exploratory analysis of different respondent groups’ opinions.

All free text responses were coded by the project lead, broadly using a framework method^34^ but remaining ‘close to the raw data’,^35^ that is, retaining information on respondents’ feedback, without drawing comments into too few themes. Coding of principle feedback questions was first deductive, recording whether each comment was about clarity, feasibility, acceptability, novelty or something else. All comment responses (from all open questions across the survey) were then coded inductively, starting with granular categorisation based on each comment’s content, then combining these granular categories where there was sufficient overlap. Finally, we recorded whether each comment suggested a change to the existing principles. Coding was double-checked by another steering group member (R.L. or L.C.-H.) for a randomly selected 10% of responses.

We agreed in advance how the responses should affect the draft PeRSEVERE principles. This included a rule that if the median responses to the questions about clarity and acceptability indicated at least ‘agreement’ (i.e. a median of at least 4/5) within each of the primarily professional and non-professional groups, then the current principle wording would not need substantial change. If this threshold was not met, we would use respondents’ feedback to agree on suitable updates. Lack of agreement with the feasibility and novelty questions without concerns about clarity and acceptability would not automatically imply wording changes. Instead, these lower scores would be considered in the interpretation and dissemination of our results, including in the planned implementation guidance. We agreed to consider all feedback, regardless of how many respondents made a given suggestion or the overall levels of support for the related principle. We agreed on a conservative approach to making changes to avoid overemphasising new feedback.

#### Other feedback during the consultation period

Feedback received via other routes (e.g. verbally after project presentations given during the consultation period) was documented, categorised and evaluated alongside survey feedback.

### Finalising the principles (stage 3)

Following survey analysis, the project lead suggested responses to all survey feedback that implied we might amend a principle. Before finalisation, the suggested changes, and proposed final principles, were shared with the steering group. A summary of the significant proposed changes was sent to members of the wider PeRSEVERE collaboration and project mailing lists (which included some survey respondents).

### Implementation guidance (stage 4)

The project lead drafted guidance for implementing the principles using suggestions made during stage 1, responses to the consultation, approaches suggested or implied in the PeRSEVERE principles themselves and evidence from non-systematic literature reviews. We also conducted a piloting exercise within the UKCRC Registered CTU Network. For this exercise, we asked volunteering CTUs to review the PeRSEVERE principles and comment on the extent to which they currently follow each principle (on a 5-point scale from ‘very closely’ to ‘not … at all’), how they currently follow the principles and how they might amend current practices to follow them more closely.

Before finalisation, the drafted implementation guidance was circulated for feedback to the PeRSEVERE steering group, representatives of the piloting CTUs, members of the stage 1 working groups and members of relevant Registered CTU Network operations groups.^36^

### Patient and public involvement

Public contributors were involved in PeRSEVERE from stage 1 onwards, to ensure a strong non-researcher presence in steering the project, given its considerable potential implications for patients and the public. At most there were seven contributors on the project steering group, who also met periodically as a standalone public contributor group. More information on the involvement activities, and a completed GRIPP2 checklist,^37^ are available as Supplementary Materials.

## Results

### Initial principle development (stage 1)

Initial development resulted in 16 principles in four domains: ‘overarching’ principles (underpinning the overall approach; coded with an ‘O’, e.g. principle O1), ‘study development and participant information’ (‘D’), ‘data management and monitoring’ (‘M’) and ‘study reporting’ (‘R’).

Key amendments during stage 1 included principles on sharing trial results with participants who stop taking part and trial monitoring.^38^ We also refined our intended level of detail (to address more about *what* should happen than *how*) and scope (e.g. to mostly exclude issues of biological sample storage).

### Consultation (stage 2)

There were 280 consultation survey responses between 11 May and 27 August 2021. Table 1 summarises respondents’ characteristics. We had responses representing all 20 roles, 22 research areas and 18 research types in our category lists (see Supplementary Files), which we had intended to be as exhaustive as possible. One additional response was excluded as a likely duplication.

**Table 1.** Consultation survey respondent characteristics.

Characteristic	n (%)^a^
Relationship to research (‘Which of the following applies best to you?’)	
I am a research professional/I am involved in research as part of my job	167 (60)
I am a patient, carer or member of the public and I am involved in research through patient and public involvement (PPI) work	61 (22)
I am a patient, carer or member of the public and I have no involvement in research in any capacity except as a potential/actual research participant	37 (13)
Unsure/hard to say	12 (4)
[Missing response]	3 (1)
Have you ever taken part in research as a participant?	
Yes	195 (70)
No	78 (28)
Not sure	5 (2)
[Missing response]	2 (1)
If you have been a research participant, did you stop/have you stopped taking part in any elementsof the research early?	
Yes	25 (9)
No	168 (60)
Not sure	3 (1)
Not applicable	80 (29)
[Missing response]	4 (1)
Had you heard of the PeRSEVERE project before you heard about this survey?	
Yes, and I have already been involved in developing the PeRSEVERE principles	10 (4)
Yes, I had heard of it but I have not been involved in any way before now	46 (16)
No	222 (79)
[Missing response]	2 (1)
Role (‘Which of these apply to you? [Tick all that apply]’) [Top 5 responses only]	
Involved in day-to-day research management (e.g. trial manager)	74 (26)
Ethics committee member	60 (21)
Patient and public involvement contributor	53 (19)
Patient/carer/member of the public without a professional research role	50 (18)
Involved in research data management (including data entry or data cleaning)	39 (14)
[Missing response]	3 (1)
Therapeutic areas of interest (‘Which of these are relevant to you, either as a researcher or a patient? [Tick all that apply]’) [Top 5 responses only]	
Cancer	82 (29)
Diabetes	66 (24)
Heart or circulation problems (e.g. high blood pressure or stroke)	57 (20)
Mental health	55 (20)
None of the above / none in particular / not sure	46 (16)
[Missing response]	9 (3)
‘Which of the following do you have experience of working with and/or participating in? (Tick all that apply)’[Top 5 responses only]	
Clinical trials of medicines (also known as ‘CTIMPs’)	169 (60)
Questionnaire-based health research (e.g. surveys about health)	134 (48)
Late-phase clinical trials (phase II, III or IV studies)	127 (45)
Complex intervention studies	92 (33)
Medical device studies	91 (33)
[Missing response]	3 (1)
‘If you have or have had a professional role in research, how long have you had/did you have this for?’	
0-5 years	31 (11)
6-10 years	37 (13)
11-20 years	67 (24)
21+ years	53 (19)
Not applicable	81 (29)
[Missing response]	11 (4)
‘If you have or have had a professional role in research, what sector was/is this in?’	
Public sector / academia	139 (50)
Commercial sector / pharmaceutical company	9 (3)
Both public and commercial sectors	34 (12)
Other / hard to say	2 (1)
Not applicable	81 (29)
[Missing response]	15 (5)
‘If you are a patient who contributes or has contributed to patient and public involvement, how long have you done/did you do this for?’	
0-5 years	55 (20)
6-10 years	21 (8)
11-20 years	17 (6)
21+ years	8 (3)
Not applicable	150 (54)
[Missing responses]	29 (10)
‘Which country are you mainly based in?’	
The United Kingdom	258 (92)
Australia	7 (2)
Italy	2 (1)
Singapore	2 (1)
Germany	1 (<1%)
New Zealand	1 (<1%)
Spain	1 (<1%)
The United States	1 (<1%)
Vietnam	1 (<1%)
[Missing response]	6 (2)
Age	
Younger than 30 years old	8 (3)
30-45 years old	78 (28)
46-65 years old	127 (45)
66+ years old	62 (22)
[Missing response]	5 (2)
Gender	
Male	81 (29)
Female	187 (67)
Neither of the above categories	1 (<1%)
Prefer not to say	6 (2)
[Missing response]	5 (2)
Ethnicity	
Asian	8 (3)
Black	0 (0)
Mixed or multiple ethnicities	6 (2)
White	253 (90)
None of the above categories	2 (1)
Prefer not to say	7 (3)
[Missing response]	4 (1)
‘Is English your first language?’	
Yes	263 (94)
No	12 (4)
Not sure / hard to say	0 (0)
[Missing response]	5 (2)

a Some of the percentages do not add up to 100% due to rounding.

In total, 269 respondents (96%) said they ‘totally’ or ‘mostly’ agreed with our key messages. Around 70% of respondents gave detailed feedback on the principles.

Table 2 provides median scores for each question about each drafted principle, overall and within the main respondent groups. More detail is available in the Supplementary Materials, including the numbers for each point on the five-point scale. Our prospective threshold about the responses on clarity and acceptability was met (median ≥4 in both categories, within both respondent groups), meaning it was not necessary to make major changes to the existing principles. The number of respondents disagreeing or strongly disagreeing for these two attributes was low (<2% for each attribute across all principles).

**Table 2.** Median scores for each question for all draft principles, overall and within the main groups of respondents.

	This principle is clear and easy to understand(median scores^a^)			I can see how this principlecould be put into practice(median scores^a^)			I agree with what thisprinciple says (median scores^a^)			This principle already reflectsmy experience of running and/ortaking part in research (medianscores^a^)			Conclusion
Draft principle title^b^	A^c^	P^c^	NP^c^	A^c^	P^c^	NP^c^	A^c^	P^c^	NP^c^	A^c^	P^c^	NP^c^	
O1: participation can stop, reduce or change	5	5	5	5	5	**4**	5	5	5	**4**	**4**	**4**	Clear and acceptable; some uncertainty about how to put it into practice
O2: the more data, the better	5	5	5	5	5	5	5	5	5	**4**	**4**	**4**	Clear and acceptable
O3: losing contact	5	5	5	5	5	5	5	5	5	**4**	**4**	**4**	Clear and acceptable
O4: continuing data collection	5	5	5	**4**	5	**4**	5	5	5	**4**	**4**	**4**	Clear and acceptable; some uncertainty about how to put into practice
O5: retaining data	5	5	5	5	5	5	5	5	5	**4**	5	**4**	Clear and acceptable
D1: protecting study integrity by design	5	5	5	**4**	5	**4**	5	5	5	**4**	**4**	**4**	Clear and acceptable; some uncertainty about how to put into practice
D2: protocol content	5	5	5	5	5	5	5	5	5	**4**	**4**	**4**	Clear and acceptable
D3: statistical planning	5	5	5	5	5	5	5	5	5	**4**	**4**	**4**	Clear and acceptable;
D4: participant information about stopping participation	5	5	5	5	5	5	5	5	5	**4**	**4**	**4**	Clear and acceptable
D5: participant information about losing contact	5	5	5	5	5	5	5	5	5	**4**	**4**	**3**	Clear and acceptable
D6: proactive discussions about participation	5	5	5	5	5	5	5	5	5	**4**	**4**	**3.5**	Clear and acceptable
D7: training and support	5	5	5	5	5	5	5	5	5	**4**	**4**	**3**	Clear and acceptable
M1: informative data collection about participation changes	5	5	5	5	5	5	5	5	5	**4**	**4**	**3**	Clear and acceptable
M2: monitoring	5	5	5	5	5	5	5	5	5	**4**	**4**	**3**	Clear and acceptable
R1: consistent and complete reporting	5	5	5	5	5	5	5	5	5	**4**	**4**	**3**	Clear and acceptable
R2: study results for all	5	5	5	5	5	5	5	5	5	**4**	**4**	**3**	Clear and acceptable

a 5 = Strongly agree, 4 = Agree, 3 = Not sure, 2 = Disagree, 1 = Strongly disagree. Missing responses excluded; values imputed only where free text comments unambiguously implied a response (only 1 value imputed). Overall proportion of missing responses is around 2%. Bold formatting is used to highlight medians less than 5.

b See the consultation survey text in Supplementary Information for the full text that was displayed to survey respondents. The reference to each principle in this table comprises the short title and the principle code. The letter in each code reflects the domain, as further explained in the text: ‘O’ for overarching principles, ‘D’ for study development including participant information, ‘M’ for data management and monitoring and ‘R’ for ‘study reporting’.

c A = median for all respondents; P = median for those primarily identifying as research professionals; NP = median for those primarily identifying as not research professionals.

Exploratory subgroup analysis suggested that groups less likely to support the principles were ethics committee members (87% saying at least ‘agree’ vs 94% for other respondents) and those with pharmaceutical industry experience (88% saying at least ‘agree’ vs 93% for other respondents). Further work would be needed to establish whether these observed differences reflect true variation between these groups’ views.

Respondents left 1071 survey comments, and there were 15 additional points of feedback outside the survey (see Supplementary Materials for full summary of free text feedback). Some recurring topics included uncertainty about how to implement the principles, worries about the risk of coercion or pressuring research participants, worries about possible negative effects on trial integrity (i.e. to do with ‘partial participation’) and difficulty applying the principles to some trial types (e.g. early phase trials).

Respondents’ views on currently used terminology (e.g. ‘withdrawn’ and ‘drop-out’) were mixed: while most respondents felt current terminology is ‘very clear’ (14%, n = 39) or ‘somewhat clear’ (39%, n = 109), a substantial minority (40%, n = 113) considered it ‘somewhat unclear’ or ‘very unclear’. Respondents felt our suggested terminology was clear and easy to understand (79% ‘strongly agree’ or ‘agree’, n = 223) and an improvement on currently used language (74% ‘strongly agree’ or ‘agree’, n = 208). Our suggestions are available in the resources section of our project website.^39^

### Final principles and implementation guidance (stages 3 and 4)

Major changes made to the principles after the consultation are summarised in Table 3. There are 17 finalised principles: seven in the ‘overarching’ domain, six for ‘study development and participant information’, and two each for ‘data management and monitoring’ and the renamed ‘study analysis and reporting’. The principles were made public in April 2022. The key messages from the final set of principles are shown in Table 4, with full text available in the Supplementary Materials, and on our website with explanatory guidance.^40^

**Table 3.** Summary of major changes made to draft PeRSEVERE principles following consultation survey.

Draft principle (‘title‘) / section^a^	Final principle (‘title’) / section^a^	Major changes following consultation survey	Rationale
Introductory text and scope	Introductory text and scope	Highlighting the particular importance of public contributors, statisticians and methodologists	Various survey comments suggested these roles were particularly important in managing clinical trial participation changes. We agreed to highlight these in our guidance, without undervaluing the role that other team members can play.
		More explicitly accommodating flexibility in applying our principles depending on the type of study	This was raised as a concern in the survey, and it did match our intention to provide broad principles to guide practice, rather than prescriptive rules.
		Changes to scope and limitations: - Confirming that issues of lack of, loss of and regaining of capacity to consent are mostly out of our project’s scope; - Addressing the issue of participants dying during a study and how this relates to our project; - Text explaining the relevance to our project of consent in the context of privacy and confidentiality.	Clarifications in response to uncertainty or misunderstanding in the survey responses.
O1 Participation can stop, reduce or change	O1 Participation can stop, reduce or change	No major changes	
N/a	O2 Participants decide how their participation changes	New principle	This was added to address various comments in the consultation that other principles might be coercive. Adding this in confirmed that choices about the extent of participation changes are primarily made by participants (with the caveat about others making decisions to protect participants’ safety, where this applies). This also linked our principles better with existing principles of Good Clinical Practice, i.e. about informed consent being informed and voluntary.
O2 The more data, the better	O3 The more data, the better	No major changes, but feedback for this principle led to the addition of the new principle O2 (above)	
O3 Losing contact	O4 Losing contact	Changes to explanatory text: - Explaining more clearly why loss of contact is different from an explicit wish to stop participating (because in the former case, the participant’s wishes can only be assumed, and assumptions may not be right) - Confirming that any further contributions must be for the purposes of the study that the participant consented to, i.e. not a green light for researchers to collect data for other purposes. - Confirming that further contact must be done sensitively and without making the participant feel pressured. - Mentioning the potential for a simple way for participants to say they want all contact to stop, without having to speak to anyone about it.	The principle ‘O3’ (and related one ‘D5’ about information for participants) was difficult for some survey respondents to accept in their current form. We agreed these were still important to include in our principle set but various amendments were made to strengthen them.
O4 Continuing data collection	O5 Continuing data collection	Extending the suggested approach to continuing data collection to potentially apply to other aspects of participation – meaning those could also continue until a participant says they want them to stop.	Amended in response to survey feedback, and because we agreed the same rationale could apply to other ‘low burden’ aspects of trial participation. This can be useful, as there are practical challenges that come up in practice, particularly around things like biological samples – i.e. if a participant has given a general wish to stop participating in a clinical trial but said nothing about what they want regarding their biological samples, what should happen? This principle would mean the biological samples could still be used for the research, as long as certain conditions were met (as described in the principle’s explanatory text). We made other changes to the explanatory text, including about ensuring the approach is applied transparently and conservatively/cautiously.
O5 Retaining data	O6 Retaining data	No major changes	
D1 Protecting study integrity by design	D1 Protecting study integrity by design	No major changes	
D2 Protocol content	D2 Protocol content	No major changes	
D3 Statistical planning	R1 Analysing studies with participation changes	Revised focus of existing principle; the existing D3 text was expanded in scope to cover validity of analysis, with statistical planning as an important supporting element.	The previous draft principle D3 covered statistical planning, but it emerged from the survey that the existing principles had not covered the need to ensure participation changes are accounted for in study analysis. The omission may be explained by the fact that this requirement is already well established and well addressed by existing guidance (e.g. by the ICH guidance on estimands and by a significant amount of published statistical literature). However, it is an essential consideration, so it belongs in our principles.
D4 Participant information about stopping participation	D3 Participant information about stopping participation	Various changes to the explanatory text: - To explain more clearly what ‘balanced’ information means, linking more directly to the new principle ‘O2’ about the extent of participation changes being participants’ informed and voluntary choice to make; - Adding content to say that potential participants should also get ‘balanced’ information about giving a reason for stopping, so that they can make an informed decision about that as well; - Content added to address the question of how much detail potential participants should get about their options for reducing or changing their participation later on – the suggestion is that it might be unhelpful or confusing to provide lots of options at that stage, but researchers can have these available to mention to participants who are struggling later on.	Clarifications and amendments to address concerns raised in the survey.
D5 Participant information about losing contact	D4 Participant information about losing contact	No major changes	
D6 Proactive discussions about participation	D5 Encouraging dialogue	Revised focus of existing principle	Various comments on the survey questioned the idea of researchers/research staff being expected to have frequent or regular discussions with participants about their ongoing participation. There were concerns about the feasibility of this (particularly given the range of different follow-up schedules in different studies) and about it being off-putting for participants. The principle was therefore amended to be broader and more flexible, emphasising the need for researchers and participants to both feel that they can raise issues with participation, rather than always discussing them.
D7 Training and support	D6 Training and support	No major changes	
M1 Informative data collection about participation changes	M1 Informative data collection about participation changes	No major changes	
M2 Monitoring	M2 Monitoring	Amended the scope of principle to include individual participant-level monitoring.	Several comments in the survey suggested that the principle M2 on monitoring might also apply to individual-level review as well as review of trends (some people even interpreted ‘monitoring’ to mean this and did not understand the rest of the principle as a result). We agreed this was a useful suggestion, so we added new content about this. It may not apply to every study, so instead it says that researchers should consider whether or not individual reviews are necessary, based on the risks and nature of the study. The reference ‘to checking participation changes have generally been handled in ways that do the best by individual participants and by the study’ means, in other words, checking that the PeRSEVERE principles have been applied.
R1 Consistent and complete trial reporting	R2 Consistent and complete trial reporting	No major changes	
R2 Study results for all	O7 Information after stopping participation	Revised focus of existing principle; principle revised as an ‘overarching’ principle	This new principle is based on a previous draft principle R2, about sharing results with participants. It became clear via the consultation response and through ongoing work related to PeRSEVERE that there are other sorts of information that participants who stop taking part early might want or need. We therefore agreed to expand the scope of the principle to incorporate all sorts of information.

a The reference to each principle in this table comprises the short title and the principle code. The letter in each code reflects the domain, as further explained in the text: ‘O’ for overarching principles, ‘D’ for study development including participant information, ‘M’ for data management and monitoring and ‘R’ for ‘study reporting’.

**Table 4.** Final PeRSEVERE principles (key messages only).

Principle code^a^	Principle ‘title’	Key message^b^
Overarching Principles		
O1	Participation can stop, reduce or change	Everyone running or taking part in studies should be aware that participants may choose to change, reduce or stop their participation after they agree to join the study.
O2	Participants decide how their participation changes	The nature and extent of participation changes should be the participant’s decision to make, within the limits of what is possible for a given study. Their decision should be informed and freely given.
O3	The more data, the better	Everyone running or taking part in studies should be aware that collecting as much as possible of a study’s planned data can help a study reach a clear and reliable conclusion.
O4	Losing contact	Loss of contact between a participant and researchers should not be considered the same as a participant saying that they want to stop study participation.
O5	Continuing data collection	Study data collection should continue until a study participant explicitly tells researchers that they want it to stop.
O6	Retaining data	Data collected for a study up to the point a study participant stops providing data should be used in the study analysis and kept with the other study data until the study is over.
O7	Information after stopping participation	Stopping participation early does not affect participants’ right to receive study-related information later on, if they want to receive it or if it could be important for them to have.
Study Development and Participant Information		
D1	Protecting study integrity by design	Studies should be designed and resourced to allow data collection to continue wherever possible, particularly for study outcome data.
D2	Protocol content	Study protocols should include clear instructions on how participation changes should be practically managed.
D3	Participant information about stopping participation	Before participants agree to take part in a study, they should receive clear and balanced information about what will happen if they want to stop participating.
D4	Participant information about losing contact	Participants should be informed, before they consent to join a study, about what will happen if contact is lost during the study.
D5	Encouraging dialogue	Throughout each study, researchers should make reasonable efforts to check that participants are still willing and able to take part. Researchers should be prepared to discuss possible changes to participation, where these might allow participants who are still willing to make a contribution to the study to do so.
D6	Training and support	Everyone involved in running studies should be trained and supported to manage participation changes for the good of both the participants and the study.
Data Management and Monitoring		
M1	Informative data collection about participation changes	Data about study participation changes should be recorded in a standardised way and include enough detail to usefully inform study management, analysis and reporting.
M2	Monitoring	All those responsible for running and overseeing a study should, at appropriately regular intervals, review summarised data about participation changes in the study.
Study Analysis and Reporting		
R1	Analysing studies with participation changes	When participation changes mean that not all the study data has been collected as planned, researchers should analyse the study in ways that give the best chance that the study will still have reliable results.
R2	Consistent and complete reporting	End of study reporting of participation changes should be done consistently within a study, showing any changes in level of participation, preferably split by treatment group.

a The reference to each principle in this table comprises the short title and the principle code. The letter in each code reflects the domain, as further explained in the text: ‘O’ for overarching principles, ‘D’ for study development including participant information, ‘M’ for data management and monitoring and ‘R’ for ‘study reporting’.

b The full text of each principle, omitted here to save space but available in the Supplementary Materials, provides more detail, including about conditions and caveats that go with some of these key messages. Further detail about how we suggest the principles can be implemented fairly and appropriately is available on our website at https://persevereprinciples.org/the-persevere-principles/.

Eight CTUs participated in our ‘piloting’ exercise. The CTUs reported, on average, ‘somewhat closely’ following the PeRSEVERE principles. They reported ‘very closely’ following the principles about retaining data (‘O6’), analysing trials with participation changes (‘R1’) and trial reporting (‘R2’). They indicated the lowest adherence to principles about protocol content clarity (‘D2’), patient information about losing contact (‘D4’) and information after stopping participation (‘O7’).

The final implementation guidance (available on our project website)^41^ contains comprehensive suggestions for applying the PeRSEVERE principles in various areas of clinical trial conduct and CTU process.

## Discussion

Our final set of principles accounts for real-life complexities in how trial participation can change and provides comprehensive guidance about the management of different types of participation change. Our principles aim for maximal adherence to the GCP aims of protecting participants’ interests and trial integrity,^2^ with priority given to participants’ interests where there is conflict but with detriment to trial integrity otherwise avoided. Our principles cover clinical trial design, setup, conduct, analysis and reporting and are designed to be flexible enough to apply to most or all clinical trials. These ideas may apply to other clinical research settings, although further exploration of this is needed.

Our consultation indicated good support for our draft principles from across the trials community, including research professionals and public contributors. We nonetheless used respondents’ feedback to strengthen our outputs. Many respondents felt the principles aligned somewhat with what already happens in practice, and we suggest this lends further support to our principles. This alignment could be because research professionals have tended to reach similar conclusions to ours regarding how GCP ideas apply to practical complexities of managing participation changes. However, there is still limited detailed guidance on managing participation changes in trials (although we note that content on ‘changing consent’ in the World Health Organization’s updated clinical trials guidance aligns well with our recommendations).^42^ We still observe some of the same variability and uncertainty in practice that motivated the PeRSEVERE project, including on the fundamental understanding and communication of complexity in participation changes (as per our principle ‘O1’). We hope our project will replace reliance on individuals’ judgement with an established, agreed best practice.

This work has provided an opportunity to harmonise thinking across different interest-holding groups, which might otherwise have reached slightly different conclusions. Some previous publications have relied on positions defined in the PeRSEVERE principles in their methods or conclusions. For example, prior publications have mentioned that potential participants should be informed about the positive consequences of greater retention in trials.^27,28^ Our work strengthens the findings from previous projects by highlighting the ethical case supporting such positions and what caveats we might place around them.

The PeRSEVERE principles have potential implications for everyone associated with clinical trials, in any capacity. Table 5 highlights the specific actions that certain groups might take. Our website includes introductory briefing notes for different interest-holding groups and example scenarios illustrating the principles’ benefits (see example in the Supplementary Materials).

**Table 5.** Suggested steps different (non-mutually exclusive) interest-holding groups might take in response to the PeRSEVERE principles.

Interest-holding group	Suggested steps they might take in response to the PeRSEVERE principles^a^
All those who design, conduct or analyse clinical trials and other research studies	Use the PeRSEVERE principles as and when required, perhaps considering them as a toolkit – choosing the required tool(s) for the situation. This could be when planning new studies – to ensure adequate preparation for possible participation changes – or during ongoing studies, to help manage more complex or unexpected scenarios. Remember in your own work PeRSEVERE’s main, underpinning message – that research participation can stop, reduce or change – and encourage others to do the same, including in their use of clear language.
Those responsible for laws and guidance about clinical trials and other research	Ensure laws and guidance allow for the real-life complexity around participation changes, rather than presenting participation as a binary condition (addressing PeRSEVERE principle O1).
Research regulators, funders and oversight bodies	Rebalance the tendency to prioritise incentives for research recruitment by increasing the importance given to retention.^43^ This would support PeRSEVERE principles O3, about collecting as much planned trial data as possible, and D1, about planning to continue data collection for as long as participants are willing. Consider whether reporting templates might need to be updated to encourage better-quality data about participation changes (addressing PeRSEVERE principle M1).
Research funders	Recognise that follow-up of participants who reduce or change their participation (as opposed to stopping entirely) can be more complicated than follow-up of participants who do not change participation. It may sometimes be necessary, therefore, for some additional funding to be allocated to this activity (addressing PeRSEVERE principle D1). It is possible that some of our recommendations could lead to improved trial retention, leading to efficiencies elsewhere over time.
Research ethics committee members	Continue to hold researchers to account over how they propose to prepare for and manage participation changes in their research, while also appreciating the balance to be struck between participants’ rights to stop taking part in research and the need to establish exactly how participants’ level of participation should change.
Protocol template owners	Ensure protocol templates are designed to cater for complexities around participation changes, including to allow for change and reduction in participation rather than just stopping, and to cover loss of contact and stopping of aspects of participation by someone other than the participant (addressing PeRSEVERE principle D2). Existing templates and guidance, such as those provided by the UK Health Research Authority,^44^ the SPIRIT statement^45^ and ICH’s proposed M11 template,^46^ do address some of our recommendations but not all. Our website now includes template wording and structure for clinical trial protocols.^47^
Organisations that provide training and support to research staff	Ensure the training provided – particularly to those with participant-facing roles – is adequate to support those individuals in dealing with real-life complexities of managing participation changes, including to uphold participants’ rights *and* trial integrity as far as possible (addressing PeRSEVERE principle D6).

a The reference to each principle in this table comprises the short title and the principle code. The letter in each code reflects the domain, as further explained in the text: ‘O’ for overarching principles, ‘D’ for study development including participant information, ‘M’ for data management and monitoring and ‘R’ for ‘study reporting’.

### Recurring ideas

Some recurring ideas permeate the PeRSEVERE principles. First is the simple need to acknowledge (as per our principle ‘O1’ and as similarly noted by others, although mostly in contexts other than clinical trials)^48–52^ that trial participation changes can be complex, and everyone – including trial participants, if possible – should be aware of this potential complexity. Use of clear language that describes how participation has changed in any given situation should follow from this acknowledgement.

A potentially more challenging idea is that of ‘presumed ongoing consent’ in some limited cases, meaning trial activities continue until participants specifically say they want those to stop. For example, if a participant says they want to stop attending trial-specific clinic visits, then those should stop, but data collection from routine data sources can continue (if the data are relevant to the trial outcomes) unless and until the participant says they want it to stop. Some consultation respondents raised concerns about principles that rely on this presumed ongoing consent idea, including around fairness and transparency from participants’ perspectives. While we acknowledge these concerns, we suggest the approach can be a suitable way to find a balance between recognising the consent previously given, respecting the update to that consent and not harming research objectives unnecessarily. Presumed ongoing consent comes with several conditions, which somewhat resemble previous recommendations around how to justifiably treat participants’ lack of ‘dissent’ as ongoing willingness.^53^ This includes being clear to potential participants about such approaches before they agree to join the trial (i.e. that they will need to specifically say they want the affected activities to stop, rather than it being implied), and expecting research staff to make reasonable efforts to establish how participants want their participation to change. In theory, it might be rare to need to rely on presumed ongoing consent, if trial processes are adequate to fully elicit participants’ wishes. However, where justified, we suggest this approach should be applied for the benefit of research integrity and therefore future patients.

A final recurring idea is about competing priorities. This is already addressed by the GCP principle that prioritises participants’ interests over those of science and society.^2^ Another potential conflict can arise between participants’ freedom to stop taking part without restriction and the importance of participants’ level of participation not changing more than dictated by their wishes (and therefore, the need for research staff to know what participants’ wishes are). On one hand, we should avoid the assumption that any indication of doubt by a participant means all aspects of participation must stop. On the other hand, processes aimed at establishing how a participant’s level of participation should change cannot be a barrier to them ending their participation.^52–54^ For example, it is not ethical to insist that participants complete a form to indicate their wishes before they stop taking part. We recognise from our consultation that some may not agree with our recommendations and feel the existing right to withdraw consent is at risk of being diluted. This is not the intention behind the PeRSEVERE principles, and we stop short of echoing suggestions to limit participants’ rights to withdraw consent in trials or other contexts.^55–58^ We fully accept there will be different views on where the fulcrum should lie in striking this sort of balance, but we hope that all can at least accept that there is a balance to strike. In other words, where a participant’s level of participation will change because their wishes have changed, reducing that level more than is justified by those wishes is bad for trials and for participants.

### Strengths and limitations

Our deliberative, inclusive process, incorporating contributions from several hundred individuals with different backgrounds and experiences, has helped address our aim of developing comprehensive, acceptable recommendations on this complex topic. Although we recognise that formal consensus methodology may have produced different results, we were not aiming to demonstrate consensus, only to ensure our outputs were acceptable to the clinical trials community as far as possible.

We did not conduct a systematic review for existing recommendations on this topic. However, our collective knowledge of the existing literature, and the results of a related scoping review,^59^ give assurances that we have a relatively complete view of the literature. It was not feasible for us to conduct more than one consultation round, although we did share the planned major changes to our outputs with some survey respondents via the project mailing list. Although we had some non-UK responses, these were in a small minority. A large majority of our respondents identified themselves as White ethnicity. We cannot judge whether this reflects a problem in how we conducted our survey or a wider issue of a lack of diversity in research, but in any case, it means we cannot know if individuals from other ethnic groups would have different feedback. We accept that our work originates in the United Kingdom and so may not apply in the same way in other jurisdictions, cultures or healthcare systems. Further work is therefore needed to assess views on managing participation changes in a more diverse range of people, contexts, countries and cultural settings. Further work to generate evidence on how best to address our principles (e.g. standardised participant information sheet wording or data collection standards) would also be worthwhile, as would testing the effects of adherence to our principles on, for example, the availability of trial outcome data.

## Conclusion

We carried out a deliberative, inclusive process to establish principles for how participation changes should be managed in clinical trials. Our final principles are more comprehensive and reflective of real-life complexity than most current ethical and regulatory guidance but still align with this guidance. Our recommendations are relevant to everyone associated with clinical trials in any capacity, and we expect their implementation will lead to improved standards in clinical trials, and a better experience for participants, in trials worldwide. We acknowledge the limitations of our project’s origins and breadth of input, and we encourage others to explore the application of these ideas in other settings and to generate empirical evidence to support best practice in this area.

## Supplemental Material

sj-docx-1-ctj-10.1177_17407745251344524 – Supplemental material for Standardising management of consent withdrawal and other clinical trial participation changes: The UKCRC Registered Clinical Trials Unit Network’s PeRSEVERE project Supplemental material, sj-docx-1-ctj-10.1177_17407745251344524 for Standardising management of consent withdrawal and other clinical trial participation changes: The UKCRC Registered Clinical Trials Unit Network’s PeRSEVERE project by William J Cragg, Laura Clifton-Hadley, Jeremy Dearling, Susan J Dutton, Katie Gillies, Pollyanna Hardy, Daniel Hind, Søren Holm, Kerenza Hood, Anna Kearney, Rebecca Lewis, Sarah Markham, Lauren Moreau, Tra My Pham, Amanda Roberts, Sharon Ruddock, Mirjana Sirovica, Ratna Sohanpal, Puvan Tharmanathan and Rejina Verghis in Clinical Trials

sj-docx-2-ctj-10.1177_17407745251344524 – Supplemental material for Standardising management of consent withdrawal and other clinical trial participation changes: The UKCRC Registered Clinical Trials Unit Network’s PeRSEVERE project Supplemental material, sj-docx-2-ctj-10.1177_17407745251344524 for Standardising management of consent withdrawal and other clinical trial participation changes: The UKCRC Registered Clinical Trials Unit Network’s PeRSEVERE project by William J Cragg, Laura Clifton-Hadley, Jeremy Dearling, Susan J Dutton, Katie Gillies, Pollyanna Hardy, Daniel Hind, Søren Holm, Kerenza Hood, Anna Kearney, Rebecca Lewis, Sarah Markham, Lauren Moreau, Tra My Pham, Amanda Roberts, Sharon Ruddock, Mirjana Sirovica, Ratna Sohanpal, Puvan Tharmanathan and Rejina Verghis in Clinical Trials

sj-docx-3-ctj-10.1177_17407745251344524 – Supplemental material for Standardising management of consent withdrawal and other clinical trial participation changes: The UKCRC Registered Clinical Trials Unit Network’s PeRSEVERE project Supplemental material, sj-docx-3-ctj-10.1177_17407745251344524 for Standardising management of consent withdrawal and other clinical trial participation changes: The UKCRC Registered Clinical Trials Unit Network’s PeRSEVERE project by William J Cragg, Laura Clifton-Hadley, Jeremy Dearling, Susan J Dutton, Katie Gillies, Pollyanna Hardy, Daniel Hind, Søren Holm, Kerenza Hood, Anna Kearney, Rebecca Lewis, Sarah Markham, Lauren Moreau, Tra My Pham, Amanda Roberts, Sharon Ruddock, Mirjana Sirovica, Ratna Sohanpal, Puvan Tharmanathan and Rejina Verghis in Clinical Trials

sj-docx-4-ctj-10.1177_17407745251344524 – Supplemental material for Standardising management of consent withdrawal and other clinical trial participation changes: The UKCRC Registered Clinical Trials Unit Network’s PeRSEVERE project Supplemental material, sj-docx-4-ctj-10.1177_17407745251344524 for Standardising management of consent withdrawal and other clinical trial participation changes: The UKCRC Registered Clinical Trials Unit Network’s PeRSEVERE project by William J Cragg, Laura Clifton-Hadley, Jeremy Dearling, Susan J Dutton, Katie Gillies, Pollyanna Hardy, Daniel Hind, Søren Holm, Kerenza Hood, Anna Kearney, Rebecca Lewis, Sarah Markham, Lauren Moreau, Tra My Pham, Amanda Roberts, Sharon Ruddock, Mirjana Sirovica, Ratna Sohanpal, Puvan Tharmanathan and Rejina Verghis in Clinical Trials

sj-docx-5-ctj-10.1177_17407745251344524 – Supplemental material for Standardising management of consent withdrawal and other clinical trial participation changes: The UKCRC Registered Clinical Trials Unit Network’s PeRSEVERE project Supplemental material, sj-docx-5-ctj-10.1177_17407745251344524 for Standardising management of consent withdrawal and other clinical trial participation changes: The UKCRC Registered Clinical Trials Unit Network’s PeRSEVERE project by William J Cragg, Laura Clifton-Hadley, Jeremy Dearling, Susan J Dutton, Katie Gillies, Pollyanna Hardy, Daniel Hind, Søren Holm, Kerenza Hood, Anna Kearney, Rebecca Lewis, Sarah Markham, Lauren Moreau, Tra My Pham, Amanda Roberts, Sharon Ruddock, Mirjana Sirovica, Ratna Sohanpal, Puvan Tharmanathan and Rejina Verghis in Clinical Trials

sj-pdf-6-ctj-10.1177_17407745251344524 – Supplemental material for Standardising management of consent withdrawal and other clinical trial participation changes: The UKCRC Registered Clinical Trials Unit Network’s PeRSEVERE project Supplemental material, sj-pdf-6-ctj-10.1177_17407745251344524 for Standardising management of consent withdrawal and other clinical trial participation changes: The UKCRC Registered Clinical Trials Unit Network’s PeRSEVERE project by William J Cragg, Laura Clifton-Hadley, Jeremy Dearling, Susan J Dutton, Katie Gillies, Pollyanna Hardy, Daniel Hind, Søren Holm, Kerenza Hood, Anna Kearney, Rebecca Lewis, Sarah Markham, Lauren Moreau, Tra My Pham, Amanda Roberts, Sharon Ruddock, Mirjana Sirovica, Ratna Sohanpal, Puvan Tharmanathan and Rejina Verghis in Clinical Trials

sj-pdf-7-ctj-10.1177_17407745251344524 – Supplemental material for Standardising management of consent withdrawal and other clinical trial participation changes: The UKCRC Registered Clinical Trials Unit Network’s PeRSEVERE project Supplemental material, sj-pdf-7-ctj-10.1177_17407745251344524 for Standardising management of consent withdrawal and other clinical trial participation changes: The UKCRC Registered Clinical Trials Unit Network’s PeRSEVERE project by William J Cragg, Laura Clifton-Hadley, Jeremy Dearling, Susan J Dutton, Katie Gillies, Pollyanna Hardy, Daniel Hind, Søren Holm, Kerenza Hood, Anna Kearney, Rebecca Lewis, Sarah Markham, Lauren Moreau, Tra My Pham, Amanda Roberts, Sharon Ruddock, Mirjana Sirovica, Ratna Sohanpal, Puvan Tharmanathan and Rejina Verghis in Clinical Trials

sj-pdf-8-ctj-10.1177_17407745251344524 – Supplemental material for Standardising management of consent withdrawal and other clinical trial participation changes: The UKCRC Registered Clinical Trials Unit Network’s PeRSEVERE project Supplemental material, sj-pdf-8-ctj-10.1177_17407745251344524 for Standardising management of consent withdrawal and other clinical trial participation changes: The UKCRC Registered Clinical Trials Unit Network’s PeRSEVERE project by William J Cragg, Laura Clifton-Hadley, Jeremy Dearling, Susan J Dutton, Katie Gillies, Pollyanna Hardy, Daniel Hind, Søren Holm, Kerenza Hood, Anna Kearney, Rebecca Lewis, Sarah Markham, Lauren Moreau, Tra My Pham, Amanda Roberts, Sharon Ruddock, Mirjana Sirovica, Ratna Sohanpal, Puvan Tharmanathan and Rejina Verghis in Clinical Trials
