# Supplementary material for: Standardising management of consent withdrawal and other clinical trial participation changes: The UKCRC Registered Clinical Trials Unit Network’s PeRSEVERE project
Source: Clin Trials. 2025 Jul 4;22(5):578–96. doi: 10.1177/17407745251344524 (PMC12476473; doi:10.1177/17407745251344524)
Supplement: sj-docx-2-ctj-10.1177_17407745251344524 – Supplemental material for Standardising management of consent withdrawal and other clinical trial participation changes: The UKCRC Registered Clinical Trials Unit Network’s PeRSEVERE project [file sj-docx-2-ctj-10.1177_17407745251344524.docx]

**Supplement 4: short form GRIPP2 reporting checklist and further details of patient and public involvement in PeRSEVERE project**

In order to report more completely the patient and public involvement in our project, without the constraints of the word limit in the main article, we have included some of the required GRIPP2 content within this supplement rather than in the article.

| **Section and topic** | **Item** | **Reported on page No** |
| --- | --- | --- |
| 1: Aim | Report the aim of PPI in the study | 14-15 and below |
| 2: Methods | Provide a clear description of the methods used for PPI in the study | 14-15 and below |
| 3: Study results | Outcomes—Report the results of PPI in the study, including both positive and negative outcomes | Below |
| 4: Discussion and conclusions | Outcomes—Comment on the extent to which PPI influenced the study overall. Describe positive and negative effects | Below |
| 5: Reflections/critical perspective | Comment critically on the study, reflecting on the things that went well and those that did not, so others can learn from this experience | Below |

From: [GRIPP2 reporting checklists: tools to improve reporting of patient and public involvement in research](https://researchinvolvement.biomedcentral.com/articles/10.1186/s40900-017-0062-2)

**Further details of Patient and Public Involvement in PeRSEVERE**

**Aim of PPI**

We recognised from the start of our project that it would be critical to involve patients and members of the public. The topic we were considering was key to participants’ experiences in trials, given it directly concerned their right to ‘withdraw’ from a trial and what should happen when they express a wish to reduce or stop their commitment.

We wanted patient/public input into 1) overall steering of the project, 2) the detail of the individual project parts and 3) how we could use the results of the project to change practice.

We wanted an adequate number of contributors, contributing in different ways, to provide balance (where needed) with the views of involved research professionals.

**PPI methods**

***Planning***

We developed a patient and public involvement plan before starting to recruit our full group of contributors. Two experienced public contributors already known to members of the Persevere steering group helped devise the plan.

We initially aimed to recruit as many as additional 6 people to join the steering group. We anticipated they might have different levels of involvement depending on their experiences and preferences, e.g. some people might be more involved in the steering group and others might prefer remote document review or other activities.

We aimed for at least one person with substantial experience of contributing to research projects, and with good links to existing patient/public groups and research groups. We considered that someone with a lot of experience and links could help ensure the project had an impact, as well as helping to improve the project outputs.

We aimed for the other contributors to have a range of experiences with research involvement, i.e. some more experienced than others. We planned for the contributors to be diverse, including in terms of gender, age, ethnicity, socioeconomic status and health conditions they have been affected by.

***Recruitment and selection of contributors***

We created a message to invite people to express an interest in contributing to the project. This explained what we were aiming to do and what would be involved for the participants. We confirmed that reimbursement would be available for any travel and time spent at in-person or online meetings.

We distributed the invitation via a range of different routes (either directly, or asking others to share on our behalf). This included existing patient/public networks, health-related charities and social media. The invitation was not confined to channels relating to any particular health condition, as the project was potentially relevant to research into any health condition.

We asked potential contributors to provide some information on themselves, including their motivation for being involved and, if they were happy to provide it, some information on their age, gender and ethnicity (self-described). We confirmed that this would only be to help ensure we got diverse contributions to the project, and that personal and sensitive information would not be shared with anyone other than those involved in the selection process.

After receipt of expressions of interest, the PeRSEVERE project lead arranged a time to briefly discuss the involvement opportunity with each potential contributor (n=42). This was to help explain what was involved, and also to get an understanding of how much each person had engaged with the topic of the project (i.e. if they were interested in the topic or just in taking part without necessarily having understood what the project was about).

The project lead prepared a de-identified list of the candidates to share with the two existing contributors. The list was reviewed independently by each reviewer and then we came together to select a suitable list of candidates for the group. Ultimately 5 additional contributors were selected, adding to the two existing contributors to make a public contributor group with 7 members.

All unsuccessful candidates were invited to join an ‘affiliate’ group if they wanted, meaning the project lead would send occasional updates about the project, including opportunities to help with small pieces of related work (with appropriate reimbursement). Over three quarters of the unsuccessful candidates chose to join the affiliate group.

***Mechanisms of involvement***

Public contributors were included in invitations for the main project steering group (which had approximately 20 research professional members). They also met separately to provide a different forum for discussion. The PeRSEVERE project lead chaired and facilitated all meetings.

Contributors were offered support to help them contribute effectively. This included a discussion (if they wanted) before beginning their contributions to the project, and separate time as needed, including one-to-one, to discuss or reflect on any issues outside of meetings.

Public contributors were invited to contribute to the same activities as the research professional members, i.e. guiding the overall project plan and progress and reviewing project documents and outputs.

Public contributors also contributed in some more specific ways. During the initial phase of developing our ‘PeRSEVERE principles’ (see main report for more details), work proceeded in 7 separate ‘writing groups’, each covering a separate area of trial design, conduct, analysis or reporting. We ensured that each of these groups had at least one public contributor, usually as a co-lead with one or two research professionals.

During that initial principle development phase, the steering group (including the public contributors) agreed that the principles and associated guidance should be written in accessible, plain language. One of the public contributors with a background in communication and creative writing took the lead in reviewing the existing content to suggest improvements in language.

**Results of PPI**

The public contributor group met periodically alongside the PeRSEVERE steering group during the active part of the project (2020-2022), and contributors were active and visible members of the steering group. Most of the contributors were interested and able to continue being involved up until the present (including as authors of the report about this project). While there may always be some scope to increase diversity of public contributors, the group of 7 on the contributor group were diverse in terms of ethnicity, age, gender, geographical location (within the UK) and their background, experiences and views. This was even more the case when including the affiliate group. This provided a rich source of input throughout the project.

Contributors made various tangible impacts on the direction of the project and content of our outputs. They helped devise the consultation survey and consider who to send it to in order to reach other members of the public. They helped review and interpret feedback received during the consultation and agree suitable amendments to our principles and guidance in response. They ensured the wording and format of the project outputs would be suitable to a general audience. They helped develop the project website (<https://persevereprinciples.org/>) and animation, and helped decide the focus of some of the online resources to prioritise outputs that would benefit patients and the public. Following release of the final PeRSEVERE principles and guidance, several of the contributors have voluntarily promoted the project through the various other projects and networks they are involved in. One of the contributors has also helped deliver training on PeRSEVERE via a workshop at the 2024 International Clinical Trials Methodology Conference in Edinburgh.

**Discussion, conclusions and reflections**

While it is valuable to consider the specific impact of patient and public involvement in this (and any) project, having a strong ‘patient voice’ in this project was essential, and we cannot envisage any valid version of this work without that.

Involving a good number of individual contributors (7 on the steering group and over 30 on the affiliate list), rather than relying on one or two people as some projects may have done in the past, meant there was a reduced risk of contributors’ views or concerns being ignored (consciously or unconsciously). It also meant there was a diversity of input, simply through each contributor having their own views to contribute to the discussions. In general, although some of the topics we discussed were technical (e.g. about statistical considerations around handling missing data in trials), the topic of our work is one that most people can engage with relatively easily, even on a ‘gut feeling’ level.

Contributors were able to successfully join in with steering group discussions, partly due to their being relatively numerous, but also through meeting facilitation by the PeRSEVERE project lead, who has strong experience in collaborating with public contributors. The use of a separate public contributor group allowed for building confidence to contribute and rapport between contributors, as well as being a useful separate forum to discuss any issues arising.

The fact of 4 of the original 7 contributors still being involved after 5 years reflects a good amount of continued engagement and support for the project. It was challenging to keep everyone involved for the duration, but this is also the case with the research professionals, as people’s professional commitments and availability change over time.

The affiliate list, plus additional contributors who have helped with related work (e.g. <https://doi.org/10.1186/s40900-024-00572-4>) has resulted in an informal network of public contributors who are interested in the topic of managing participation changes and experienced in thinking about the related issues. Many of the public contributors remain in touch with the PeRSEVERE project lead regarding ongoing projects and possible future work.

One particular challenge in the initial involvement was its timing. We were preparing to start involving people in February and March 2020, when the significance and implications of the Covid-19 pandemic was only just becoming clear. We had initially planned for some face-to-face involvement, but ultimately this proved impossible due to pandemic restrictions. There was therefore a shift to working only online, and this was new for many (including research professionals) and took some adjustment for all concerned. On the other hand, the sense from some of the initial discussions about the project between the project lead and potential contributors was that, in that early part of 2020, it was enjoyable to be connecting with people across the country and to be focussing on something engaging, positive and constructive in contrast to the various challenges that the pandemic and its impacts were posing.
