# Supplementary material for: Standardising management of consent withdrawal and other clinical trial participation changes: The UKCRC Registered Clinical Trials Unit Network’s PeRSEVERE project
Source: Clin Trials. 2025 Jul 4;22(5):578–96. doi: 10.1177/17407745251344524 (PMC12476473; doi:10.1177/17407745251344524)
Supplement: sj-docx-3-ctj-10.1177_17407745251344524 – Supplemental material for Standardising management of consent withdrawal and other clinical trial participation changes: The UKCRC Registered Clinical Trials Unit Network’s PeRSEVERE project [file sj-docx-3-ctj-10.1177_17407745251344524.docx]

**The tables on the pages below show the draft PeRSEVERE principle titles as they were presented within the consultation survey, ordered by overall proportion of respondents who said ‘agree’ or ‘strongly agree’ regarding our four attributes of interest: clarity, practicability, acceptability and novelty.**

**The proportions within primarily research professional and primarily non-professional respondent groups are also shown in each table, as well as the proportion of missing data and number of respondents giving each answer in the Likert-type scale questions.**

**The reference to each principle in these tables comprises the short title and the principle code. The letter in each code reflects the domain, as further explained in the text: “O” for overarching principles, “D” for study development including participant information, “M” for data management and monitoring and “R” for ‘study reporting’.**

**Table 1: PeRSEVERE principles ordered by survey respondents ratings on clarity (specifically, respondents were asked for their level of agreement with the statement “This principle is clear and easy to understand”)**

| **Principle name** | **% agree or strongly agree** | | | **% missing responses** | **Number of responses** | | | | | |
| --- | --- | --- | --- | --- | --- | --- | --- | --- | --- | --- |
|  | **All respondents** | **Primarily professional** | **Primarily non-professional** |  | **Strongly agree** | **Agree** | **Not sure** | **Disagree** | **Strongly disagree** | **Missing** |
| R2: study results for all | 97% | 98% | 96% | 1% | 151 | 43 | 2 | 1 | 0 | 2 |
| M2: monitoring | 97% | 98% | 95% | 1% | 145 | 43 | 2 | 2 | 0 | 1 |
| M1: informative data collection about participation changes | 97% | 98% | 97% | 1% | 137 | 51 | 2 | 2 | 0 | 1 |
| D5: participant information about losing contact | 97% | 98% | 95% | 2% | 138 | 47 | 2 | 1 | 0 | 3 |
| O1: participation can stop, reduce or change | 96% | 98% | 94% | 1% | 135 | 54 | 2 | 4 | 0 | 1 |
| O2: the more data, the better | 96% | 96% | 96% | 1% | 134 | 54 | 4 | 2 | 1 | 1 |
| O3: losing contact | 96% | 96% | 96% | 1% | 143 | 45 | 5 | 2 | 0 | 1 |
| D4: participant information about stopping participation | 96% | 96% | 95% | 2% | 142 | 41 | 3 | 2 | 0 | 3 |
| O5: retaining data | 95% | 96% | 94% | 0% | 131 | 56 | 5 | 3 | 1 | 0 |
| D3: statistical planning | 95% | 97% | 92% | 2% | 118 | 64 | 3 | 2 | 0 | 4 |
| D6: proactive discussions about participation | 95% | 94% | 97% | 2% | 149 | 33 | 6 | 0 | 0 | 3 |
| D7: training and support | 95% | 95% | 95% | 3% | 145 | 37 | 4 | 0 | 0 | 5 |
| R1: consistent and complete reporting | 95% | 98% | 91% | 1% | 137 | 52 | 7 | 1 | 0 | 2 |
| D2: protocol content | 94% | 94% | 95% | 2% | 129 | 51 | 7 | 1 | 0 | 3 |
| O4: continuing data collection | 93% | 94% | 92% | 1% | 129 | 54 | 6 | 6 | 0 | 1 |
| D1: protecting study integrity by design | 92% | 92% | 91% | 2% | 119 | 56 | 8 | 4 | 1 | 3 |

**Table 2: PeRSEVERE principles ordered by survey respondents ratings on practicability (specifically, respondents were asked for their level of agreement with the statement “I can see how this principle could be put into practice”)**

| **Principle name** | **% agree or strongly agree** | | | **% missing responses** | **Number of responses** | | | | | |
| --- | --- | --- | --- | --- | --- | --- | --- | --- | --- | --- |
|  | **All respondents** | **Primarily professional** | **Primarily non-professional** |  | **Strongly agree** | **Agree** | **Not sure** | **Disagree** | **Strongly disagree** | **Missing** |
| D4: participant information about stopping participation | 93% | 94% | 92% | 2% | 127 | 51 | 6 | 3 | 0 | 4 |
| O5: retaining data | 92% | 92% | 92% | 2% | 121 | 59 | 11 | 2 | 0 | 3 |
| O1: participation can stop, reduce or change | 91% | 92% | 90% | 1% | 99 | 80 | 14 | 1 | 0 | 2 |
| M2: monitoring | 91% | 92% | 89% | 2% | 129 | 47 | 13 | 1 | 0 | 3 |
| M1: informative data collection about participation changes | 90% | 90% | 90% | 2% | 113 | 61 | 12 | 4 | 0 | 3 |
| O2: the more data, the better | 90% | 90% | 89% | 1% | 101 | 75 | 14 | 4 | 0 | 2 |
| R1: consistent and complete reporting | 89% | 94% | 83% | 2% | 121 | 57 | 16 | 1 | 0 | 4 |
| D5: participant information about losing contact | 88% | 88% | 89% | 2% | 112 | 57 | 16 | 2 | 0 | 4 |
| D2: protocol content | 88% | 88% | 89% | 3% | 108 | 61 | 16 | 1 | 0 | 5 |
| O4: continuing data collection | 88% | 91% | 82% | 2% | 96 | 76 | 15 | 6 | 0 | 3 |
| D7: training and support | 87% | 88% | 86% | 4% | 121 | 46 | 15 | 2 | 0 | 7 |
| D6: proactive discussions about participation | 85% | 83% | 88% | 2% | 124 | 38 | 22 | 2 | 1 | 4 |
| D3: statistical planning | 84% | 87% | 78% | 3% | 101 | 59 | 24 | 2 | 0 | 5 |
| O3: losing contact | 83% | 84% | 82% | 2% | 105 | 58 | 26 | 4 | 0 | 3 |
| R2: study results for all | 83% | 79% | 89% | 2% | 117 | 48 | 21 | 9 | 1 | 3 |
| D1: protecting study integrity by design | 83% | 85% | 78% | 3% | 91 | 67 | 22 | 5 | 0 | 6 |

**Table 3: PeRSEVERE principles ordered by survey respondents ratings on acceptability (specifically, respondents were asked for their level of agreement with the statement “I agree with what this principle says”)**

| **Principle name** | **% agree or strongly agree** | | | **% missing responses** | **Number of responses** | | | | | |
| --- | --- | --- | --- | --- | --- | --- | --- | --- | --- | --- |
|  | **All respondents** | **Primarily professional** | **Primarily non-professional** |  | **Strongly agree** | **Agree** | **Not sure** | **Disagree** | **Strongly disagree** | **Missing** |
| R1: consistent and complete reporting | 96% | 98% | 95% | 1% | 132 | 60 | 4 | 0 | 1 | 2 |
| M2: monitoring | 96% | 95% | 97% | 1% | 140 | 45 | 5 | 1 | 0 | 2 |
| O1: participation can stop, reduce or change | 95% | 97% | 92% | 1% | 141 | 45 | 6 | 2 | 0 | 2 |
| D7: training and support | 95% | 96% | 92% | 3% | 141 | 40 | 3 | 1 | 1 | 5 |
| D4: participant information about stopping participation | 94% | 96% | 89% | 2% | 140 | 39 | 3 | 3 | 2 | 4 |
| D2: protocol content | 94% | 93% | 95% | 2% | 123 | 56 | 8 | 0 | 0 | 4 |
| R2: study results for all | 93% | 94% | 92% | 1% | 143 | 42 | 8 | 4 | 0 | 2 |
| D1: protecting study integrity by design | 93% | 94% | 91% | 3% | 125 | 52 | 5 | 3 | 1 | 5 |
| O3: losing contact | 92% | 90% | 94% | 2% | 138 | 42 | 8 | 4 | 1 | 3 |
| O5: retaining data | 91% | 94% | 87% | 2% | 123 | 56 | 10 | 3 | 1 | 3 |
| M1: informative data collection about participation changes | 91% | 91% | 92% | 1% | 132 | 44 | 10 | 4 | 1 | 2 |
| D5: participant information about losing contact | 91% | 91% | 91% | 2% | 125 | 49 | 8 | 4 | 2 | 3 |
| O2: the more data, the better | 91% | 90% | 93% | 2% | 125 | 53 | 9 | 5 | 1 | 3 |
| D3: statistical planning | 90% | 93% | 85% | 3% | 119 | 53 | 11 | 1 | 1 | 6 |
| O4: continuing data collection | 89% | 91% | 86% | 1% | 120 | 55 | 8 | 8 | 3 | 2 |
| D6: proactive discussions about participation | 89% | 87% | 92% | 2% | 136 | 34 | 16 | 2 | 0 | 3 |

**Table 4: PeRSEVERE principles ordered by survey respondents ratings on novelty (specifically, respondents were asked for their level of agreement with the statement “This principle already reflects my experience of running and/or taking part in research”)**

| **Principle name** | **% agree or strongly agree (all)** | | | **% missing responses** | **Number of responses** | | | | | |
| --- | --- | --- | --- | --- | --- | --- | --- | --- | --- | --- |
|  | **All respondents** | **Primarily professional** | **Primarily non-professional** |  | **Strongly agree** | **Agree** | **Not sure** | **Disagree** | **Strongly disagree** | **Missing** |
| O1: participation can stop, reduce or change | 74% | 78% | 66% | 2% | 74 | 71 | 29 | 15 | 3 | 4 |
| O5: retaining data | 74% | 84% | 56% | 4% | 91 | 54 | 31 | 11 | 1 | 8 |
| D4: participant information about stopping participation | 66% | 69% | 62% | 4% | 67 | 60 | 25 | 28 | 4 | 7 |
| O2: the more data, the better | 64% | 69% | 56% | 2% | 63 | 63 | 28 | 32 | 6 | 4 |
| D1: protecting study integrity by design | 64% | 72% | 48% | 5% | 61 | 61 | 30 | 25 | 4 | 10 |
| O4: continuing data collection | 64% | 70% | 54% | 3% | 64 | 61 | 41 | 20 | 4 | 6 |
| R1: consistent and complete reporting | 62% | 74% | 41% | 4% | 59 | 64 | 56 | 11 | 2 | 7 |
| O3: losing contact | 61% | 67% | 51% | 3% | 70 | 50 | 37 | 33 | 1 | 5 |
| M2: monitoring | 61% | 69% | 44% | 4% | 65 | 53 | 45 | 19 | 4 | 7 |
| M1: informative data collection about participation changes | 60% | 67% | 44% | 3% | 59 | 56 | 44 | 25 | 4 | 5 |
| D3: statistical planning | 57% | 62% | 48% | 6% | 56 | 53 | 52 | 15 | 4 | 11 |
| D6: proactive discussions about participation | 57% | 63% | 46% | 4% | 57 | 52 | 41 | 29 | 4 | 8 |
| D2: protocol content | 52% | 54% | 49% | 5% | 49 | 51 | 40 | 39 | 3 | 9 |
| D7: training and support | 51% | 56% | 43% | 5% | 55 | 43 | 51 | 29 | 4 | 9 |
| D5: participant information about losing contact | 51% | 56% | 43% | 4% | 46 | 52 | 44 | 37 | 5 | 7 |
| R2: study results for all | 49% | 51% | 47% | 4% | 55 | 43 | 39 | 47 | 8 | 7 |
