# Supplementary material for: Standardising management of consent withdrawal and other clinical trial participation changes: The UKCRC Registered Clinical Trials Unit Network’s PeRSEVERE project
Source: Clin Trials. 2025 Jul 4;22(5):578–96. doi: 10.1177/17407745251344524 (PMC12476473; doi:10.1177/17407745251344524)
Supplement: sj-docx-4-ctj-10.1177_17407745251344524 – Supplemental material for Standardising management of consent withdrawal and other clinical trial participation changes: The UKCRC Registered Clinical Trials Unit Network’s PeRSEVERE project [file sj-docx-4-ctj-10.1177_17407745251344524.docx]

The table below summarises the comments received in the consultation survey about the draft PeRSEVERE principles. Respondents were asked to rate each draft principle in terms of its clarity, practicability, acceptability and novelty, then offered an open question to provide feedback (in response to the question: “Do you have any feedback on this principle?”). The reference to each principle in this table comprises the short title and the principle code. The letter in each code reflects the domain, as further explained in the text: “O” for overarching principles, “D” for study development including participant information, “M” for data management and monitoring and “R” for ‘study reporting’.

The comments in the free text box were categorised by the same attributes as the rating questions (see further explanation below). Comments were also categorised more granularly based on the points each respondent was making. The final columns in the table show the five most frequent comments made on each draft principle. To be clear, these are not direct quotes. For example, the categorised comment in the table ‘generally agree’ would reflect a comment in the survey like ‘I agree’, but also ‘I support this’ or ‘this seems right to me’.

Some less meaningful comment categories are omitted from this top five listing, namely: comments where the intended meaning was unclear, comments that were more relevant to a different draft principle, and comments referring to comments given by the same respondent in a previous survey question (e.g. “see my previous comment”).

**Comment categorisation by attribute:**

- **Clarity** = comment on how clear the principle is or is not;
- **Practicability** = comment how easy or difficult the principle might be to put into practice;
- **Acceptability** = comment on how acceptable or agreeable the principle is;
- **Novelty** = comment on how much the principle reflects how research is already carried out in practice;
- **‘Other’** = comments not directly related to the principle or otherwise not fitting into the categories above; **comment but no comment** = ‘I have no comments’ or similar.

| **Draft principle** | **Number of respondents leaving a comment (% of those who completed the survey section)** | **Number of comments implying change needed to the draft principle or associated guidance** | **Comments categorised by attribute (more than one possible per person)** | **Most frequent five comment categories per principle** | | |
| --- | --- | --- | --- | --- | --- | --- |
|  |  |  |  | **Comment message (categorised)** | **n** | **Comment attribute and implication** |
| O1 Participation can stop, reduce or change | 76 (39%) | 26 | **Clarity:** 9  **Practicability:** 10  **Acceptability:** 37  **Novelty:** 22  **Other:** 31 | Generally agree | 10 | Acceptability, no change to principle implied |
|  |  |  |  | Flexibility in follow-up not always right | 8 | **Acceptability, possible principle change implied** |
|  |  |  |  | Agree everyone needs to recognise complexity and participant options | 8 | Acceptability, no change to principle implied |
|  |  |  |  | Research site staff/researchers do not implement this well enough | 8 | Novelty |
|  |  |  |  | Currently issues with patient information [with regard to this principle] | 6 | Novelty |
| O2 The more data, the better | 87 | 47 | **Clarity:** 8  **Practicability:** 14  **Acceptability:** 46  **Novelty:** 26  **Other:** 37 | Not usually communicated well to participants | 18 | Novelty |
|  |  |  |  | Generally support the principle | 17 | Acceptability, no change to principle implied |
|  |  |  |  | Concerns about risk of coercion or making participants feel guilty | 16 | **Acceptability, possible principle change implied** |
|  |  |  |  | Agree it is important to inform participants about value of study data, before they decide to take part | 7 | Acceptability, no change to principle implied |
|  |  |  |  | Need to consider data quality as well as completeness / more data not necessarily better | 6 | **Acceptability, possible principle change implied** |
| O3 Losing contact | 76 | 29 | **Clarity:** 3  **Practicability:** 24  **Acceptability:** 38  **Novelty:** 16  **Other:**39 | Generally supportive | 18 | Acceptability, no change to principle implied |
|  |  |  |  | If contact lost, researchers should assume the participant wants to stop taking part | 4 | **Acceptability, possible principle change implied** |
|  |  |  |  | Approach takes more time and effort | 4 | Practicability |
|  |  |  |  | Approach takes more time and effort | 4 | Practicability |
|  |  |  |  | Prospectively planned approach is not common | 4 | Novelty |
| O4 Continuing data collection | 90 | 42 | **Clarity:** 6  **Practicability:** 19  **Acceptability:** 47  **Novelty:** 14  **Other:** 51 | General support | 11 | Acceptability, no change to principle implied |
|  |  |  |  | Only applies if participant has/retains capacity to consent | 9 | **Acceptability, possible principle change implied** |
|  |  |  |  | Agree needs mentioning clearly, as part of the pre-study information | 7 | Acceptability, no change to principle implied |
|  |  |  |  | Challenging to express it clearly in participant information sheet/verbal information and avoid information overload | 6 | Practicability |
|  |  |  |  | Need better understanding/training for research sites | 5 | Other |
| O5 Retaining data | 62 | 37 | **Clarity:** 4  **Practicability:** 5  **Acceptability:** 41  **Novelty:** 17  **Other:** 34 | Agree important to explain about limits to data deletion before data is used | 7 | Acceptability, no change to principle implied |
|  |  |  |  | General support | 7 | Acceptability, no change to principle implied |
|  |  |  |  | Participants should be able to ask for data about them to be deleted in some/all cases | 6 | **Acceptability, possible principle change implied** |
|  |  |  |  | This topic is poorly communicated to participants | 5 | Novelty |
|  |  |  |  | Don't agree that data should always be made available, even for approved, bone fide research and in line with consent | 4 | **Acceptability, possible principle change implied** |
| D1 Protecting study integrity by design | 63 | 26 | **Clarity:** 7  **Practicability:** 18  **Acceptability:** 28  **Novelty:** 13  **Other:** 33 | General support | 8 | Acceptability, no change to principle implied |
|  |  |  |  | Support flexibility to accommodate participants' changing circumstances | 6 | Acceptability, no change to principle implied |
|  |  |  |  | May be difficult to put into practice (generally) | 5 | Practicability |
|  |  |  |  | Must be transparent with participants about data collection methods / participants must agree/consent to them | 4 | **Acceptability, possible principle change implied** |
|  |  |  |  | Presenting participants with options may encourage them to stop or reduce participation when they otherwise would not have | 3 | Practicability |
|  |  |  |  | Studies not usually designed for reduced participation or flexibility with follow-up | 3 | Novelty |
|  |  |  |  | Already considered best practice / acceptable by most | 3 | Novelty |
| D2 Protocol content | 66 | 11 | **Clarity:** 1  **Practicability:** 11  **Acceptability:** 25  **Novelty:** 17  **Other:** 38 | General support | 16 | Acceptability, no change to principle implied |
|  |  |  |  | Do not tend to see this covered in protocols [i.e. proposed level of detail] | 12 | Novelty |
|  |  |  |  | Patient information is not adequate about this topic / important that patient information is clear | 5 | Other |
|  |  |  |  | Difficult to think of all possibilities before a study begins, or cover them in enough detail | 4 | Practicability |
|  |  |  |  | Support recording participants' wishes and their reason for stopping if the participant gives one | 4 | Acceptability, no change to principle implied |
| D3 Statistical planning | 58 | 19 | **Clarity:** 4  **Practicability:** 19  **Acceptability:** 19  **Novelty:** 18  **Other:**28 | General support | 11 | Acceptability, no change to principle implied |
|  |  |  |  | Challenging to plan everything pre-study because too many unknowns / analysis plan will inevitably need to be updated later | 7 | Practicability |
|  |  |  |  | This is already done | 7 | Novelty |
|  |  |  |  | Sometimes analysis planning is quite limited at the start of a study / there is sometimes/usually not a plan in place at the start | 5 | Novelty |
|  |  |  |  | Difficult to accurately predict how many participants will stop taking part early | 4 | Practicability |
|  |  |  |  | Inflating the sample size to account for early stopping is commonly done | 4 | Novelty |
| D4 Participant information about stopping participation | 67 | 21 | **Clarity:** 1  **Practicability:** 18  **Acceptability:** 26  **Novelty:** 21  **Other:** 31 | Challenge to avoid real or perceived coercion | 11 | Practicability |
|  |  |  |  | General support | 7 | Acceptability, no change to principle implied |
|  |  |  |  | Support the idea that participants should be told about the impact of stopping early on the study / why it can be good for the study if more participants continue taking part in some way / why data is important |  | Acceptability, no change to principle implied |
|  |  |  |  | Concerns about coercion | 4 | **Acceptability, possible principle change implied** |
|  |  |  |  | Explanations about the importance of data collection tend to be unclear / absent | 4 | Novelty |
|  |  |  |  | Patient information already clear about participants' rights to stop taking part at any time | 4 | Novelty |
|  |  |  |  | Not done (well) at the moment | 4 | Novelty |
| D5 Participant information about losing contact | 66 | 50 | **Clarity:** 3  **Practicability:** 8  **Acceptability:** 25  **Novelty:** 11  **Other:** 45 | General support | 8 | Acceptability, no change to principle implied |
|  |  |  |  | Agree that participants need to be informed | 5 | Acceptability, no change to principle implied |
|  |  |  |  | Not usually well-communicated to patients before a study | 4 | Novelty |
|  |  |  |  | Not sure that pre-study consent is suitable approach | 3 | **Acceptability, possible principle change implied** |
|  |  |  |  | Could be challenging to explain this clearly to patients | 3 | Practicability |
|  |  |  |  | This issue is rarely considered in advance / not enough | 3 | Novelty |
| D6 Proactive discussions about participation | 69 | 55 | **Clarity:** 5  **Practicability:** 10  **Acceptability:** 40  **Novelty:** 17  **Other:** 36 | General support | 14 | Acceptability, no change to principle implied |
|  |  |  |  | Does not usually happen / not enough | 7 | Novelty |
|  |  |  |  | Research staff do not have time to do this or to deal with each participant's concerns/issues / generally not feasible | 7 | Practicability |
|  |  |  |  | May not apply to some studies | 6 | **Acceptability, possible principle change implied** |
|  |  |  |  | Repeated checking might be burdensome for participant or researcher | 5 | **Acceptability, possible principle change implied** |
|  |  |  |  | Regular checking may increase the number of participants who stop participation early | 5 | **Acceptability, possible principle change implied** |
| D7 Training and support | 60 | 17 | **Clarity:** 2  **Practicability:** 8  **Acceptability:** 32  **Novelty:** 19  **Other:** 33 | General support | 19 | Acceptability, no change to principle implied |
|  |  |  |  | Not currently done / done well | 6 | Novelty |
|  |  |  |  | Concerns that suggested approach impacts on participants' rights | 5 | **Acceptability, possible principle change implied** |
|  |  |  |  | Research sites tend to assume a participant wants to end all their involvement / do not have good understanding about different 'levels' of participation | 3 | Novelty |
|  |  |  |  | [Many comments made 2 times] | 2 | [Various] |
| M1 Informative data collection about participation changes | 64 | 20 | **Clarity:** 1  **Practicability:** 15  **Acceptability:** 24  **Novelty:** 14  **Other:** 36 | Not appropriate/acceptable to insist participants give a reason for stopping participation | 7 | **Acceptability, possible principle change implied** |
|  |  |  |  | Agree it can be useful to know why participation changes have occurred | 7 | Acceptability, no change to principle implied |
|  |  |  |  | Participants are not obliged to give a reason for stopping participation | 6 | Other |
|  |  |  |  | Challenges in designing data collection or study databases | 6 | Practicability |
|  |  |  |  | General support | 6 | Acceptability, no change to principle implied |
| M2 Monitoring | 44 | 15 | **Clarity:** 2  **Practicability:** 4  **Acceptability:** 24  **Novelty:** 12  **Other:** 18 | General support | 12 | Acceptability, no change to principle implied |
|  |  |  |  | Already done | 6 | Novelty |
|  |  |  |  | Does not happen (/enough) | 4 | Novelty |
|  |  |  |  | Resulting actions after data review must not pressure or coerce participants to continue providing data | 2 | **Acceptability, possible principle change implied** |
|  |  |  |  | Agree that reviewing information on why participation changes are happening can be informative | 2 | Acceptability, no change to principle implied |
| R1 Consistent and complete trial reporting | 41 | 11 | **Clarity:** 4  **Practicability:** 6  **Acceptability:** 18  **Novelty:** 6  **Other:** 20 | General support | 9 | Acceptability, no change to principle implied |
|  |  |  |  | Agree this supports future studies | 3 | Acceptability, no change to principle implied |
|  |  |  |  | Variable how well reporting is done / not done well enough | 3 | Novelty |
|  |  |  |  | Participants do not get feedback/results of studies they took part in | 3 | Other |
|  |  |  |  | This bundles together two distinct issues of reporting and missing data methods in analysis | 2 | **Acceptability, possible principle change implied** |
|  |  |  |  | Would be useful to have updated guidelines to cover this aspect in more detail | 2 | Other |
|  |  |  |  | Plans for handling missing data are not / should be clear in protocols and analysis plans | 2 | Other |
|  |  |  |  | Studies should have a larger sample size to allow for some participants' decisions to stop participating early | 2 | Other |
| R2 Study results for all | 82 | 23 | **Clarity:** 3  **Practicability:** 28  **Acceptability:** 41  **Novelty:** 27  **Other:** 42 | General support for the principle | 16 | Acceptability, no change to principle implied |
|  |  |  |  | Does not happen (enough) | 11 | Novelty |
|  |  |  |  | Resourcing challenges at the end-stage of a study | 9 | Practicability |
|  |  |  |  | General support for sharing/offering study results | 8 | Acceptability, no change to principle implied |
|  |  |  |  | Practically difficult (in general) / not always possible | 6 | Practicability |
