# Supplementary material for: Standardising management of consent withdrawal and other clinical trial participation changes: The UKCRC Registered Clinical Trials Unit Network’s PeRSEVERE project
Source: Clin Trials. 2025 Jul 4;22(5):578–96. doi: 10.1177/17407745251344524 (PMC12476473; doi:10.1177/17407745251344524)
Supplement: sj-docx-5-ctj-10.1177_17407745251344524 – Supplemental material for Standardising management of consent withdrawal and other clinical trial participation changes: The UKCRC Registered Clinical Trials Unit Network’s PeRSEVERE project [file sj-docx-5-ctj-10.1177_17407745251344524.docx]

**PeRSEVERE principles**

Further explanatory guidance is available at <https://persevereprinciples.org/the-persevere-principles/>.

The bold heading for each principle comprises the short title and the principle code. The letter in each code reflects the domain, as further explained in the text: “O” for overarching principles, “D” for study development including participant information, “M” for data management and monitoring and “R” for ‘study reporting’.

**O1: participation can stop, reduce or change**

Everyone running or taking part in studies should be aware that participants may choose to change, reduce or stop their participation after they agree to join the study.

All language and communication about any participation changes should be clear about exactly what has changed, reduced or stopped, and what has not.

**O2: participants decide how their participation changes**

The nature and extent of participation changes should be the participant’s decision to make, within the limits of what is possible for a given study. Their decision should be informed and freely-given.

The only exception to this is where aspects of participation need to change or be stopped by someone else in order to protect a participant’s safety or well-being.

**O3: the more data, the better**

Everyone running or taking part in studies should be aware that collecting as much as possible of a study’s planned data can help a study reach a clear and reliable conclusion.

This should be made clear to potential study participants using ethically-approved wording before they agree to take part in the study.

**O4: losing contact**

Loss of contact between a participant and researchers should not be considered the same as a participant saying that they want to stop study participation.

**O5: continuing data collection**

Study data collection should continue until a study participant explicitly tells researchers that they want it to stop.

This approach is valid and fair only if it is made clear to participants before they join the study, including how they can express a wish for stopping data collection, if that is what they want.

Researchers should also make all reasonable efforts to find out exactly which aspects of study participation a participant wants to stop, if they express the wish to stop or reduce their participation. Any further data collection must still be done in line with the informed consent that participants previously gave.

This approach can also be applied to other aspects of participation, as long as those aspects are necessary and have a relatively low impact on participants, and as long as any further activity is done according to the conditions mentioned in this principle.

**O6: retaining data**

Data collected for a study up to the point a study participant stops providing data should be used in the study analysis, and kept with the other study data until the study is over.

The data should also be made available for legitimate additional research in line with participant consent and appropriate approvals.

**O7: information after stopping participation**

Stopping participation early does not affect participants’ right to receive study-related information later on, if they want to receive it or if it could be important for them to have.

**D1: protecting study integrity by design**

Studies should be designed and resourced to allow data collection to continue wherever possible, particularly for study outcome data.

Participants should be allowed to continue participating while making less commitment to the study, where this is feasible, safe and does not negatively affect the scientific integrity of the study.

**D2: protocol content**

Study protocols should include clear instructions on how participation changes should be practically managed.

This should include, where necessary, study-specific definitions for different types of participation change expected over the time the study will take place. Researchers should decide how these participation changes affect the specific research question they want to answer.

Protocols should also include a pre-defined plan, developed with patient involvement, for appropriate actions to take if study researchers lose contact with study participants.

**D3: participant information about stopping participation**

Before participants agree to take part in a study, they should receive clear and balanced information about what will happen if they want to stop participating.

This should include information about how it is good for the study if participants can provide outcome data until the study ends.

This way, participants can make an informed choice about initial and ongoing involvement.

**D4: participant information about losing contact**

Participants should be informed, before they consent to join a study, about what will happen if contact is lost during the study.

If any participants might feel that the ways that researchers may try to get back in touch are intrusive, participants should have the chance to provide freely-given, informed consent to allow these contact methods to go ahead.

Researchers may not carry out these attempts at further contact if participants have not given consent.

**D5: encouraging dialogue**

Throughout each study, researchers should make reasonable efforts to check that participants are still willing and able to take part.

Researchers should be prepared to discuss possible changes to participation, where these might allow participants who are still willing to make a contribution to the study to do so.

Participants should be encouraged to contact the researchers at the earliest opportunity if they are experiencing difficulties with any part of the study or if their circumstances may be changing in ways that will make taking part more difficult.

**D6: training and support**

Everyone involved in running studies should be trained and supported to manage participation changes for the good of both the participants and the study.

This should be done in line with applicable regulations and include an understanding of the importance of continuing study data collection wherever possible.

Training should acknowledge that satisfying participants’ wishes for less involvement in a study may not need to result in their participation stopping altogether.

**M1: informative data collection about participation changes**

Data about study participation changes should be recorded in a standardised way and include enough detail to usefully inform study management, analysis and reporting.

Data should include, when available, meaningful information about when and why the participant has reduced or stopped their participation.

Collected data should also clearly communicate the participant’s wishes, including which elements of study participation they want to stop, and which they have agreed to continue.

**M2: monitoring**

All those responsible for running and overseeing a study should, at appropriately regular intervals, review summarised data about participation changes in the study.

This allows them to identify common issues or developing trends in study participation, and reasons for these trends, so that timely and targeted action can be taken.

Those running and overseeing studies should also consider reviewing information about the participation changes of individual study participants, if this might be important for their study.

**R1: analysing studies with participation changes**

When participation changes mean that not all the study data has been collected as planned, researchers should analyse the study in ways that give the best chance that the study will still have reliable results.

The analysis should be done using methods that are planned in as much detail as possible before the study starts, and that follow current best practice for the specific research questions in the study.

**R2: consistent and complete reporting**

End of study reporting of participation changes should be done consistently within a study, showing any changes in level of participation, preferably split by treatment group.

This helps with the assessment of the quality of the study and of the reliability of the results. It can also inform the size, design and conduct of future studies.

Methods used to handle missing data should also be described, to allow interpretation and replication of results.
