# Supplementary material for: Standardising management of consent withdrawal and other clinical trial participation changes: The UKCRC Registered Clinical Trials Unit Network’s PeRSEVERE project
Source: Clin Trials. 2025 Jul 4;22(5):578–96. doi: 10.1177/17407745251344524 (PMC12476473; doi:10.1177/17407745251344524)
Supplement: sj-pdf-6-ctj-10.1177_17407745251344524 – Supplemental material for Standardising management of consent withdrawal and other clinical trial participation changes: The UKCRC Registered Clinical Trials Unit Network’s PeRSEVERE project [file sj-pdf-6-ctj-10.1177_17407745251344524.pdf]

PeRSEVERE: PRincipleS for handling end of participation EVEnts in clinical trials REsearch

Consultation plan

V1.0, 11 March 2021

PeRSEVERE collaborative group

Contents

1. Background and Purpose 3
2. Consultation 4
2.1. Overview 4
2.2. Feedback by survey 4
2.2.1. Survey eligibility 4
2.2.2. Recruitment and consent 4
2.2.3. Survey design and data collection 5
2.2.4. Handling survey feedback 7
2.2.4.1. Data management, data cleaning and handling missing data 7
2.2.4.2. Quantitative data analysis 8
2.2.4.3. Qualitative data analysis 8
2.2.4.4. Monitoring and reporting 9
2.2.4.5. Informing further development of PeRSEVERE principles 9
2.2.5. Sample size 11
2.2.6. Ethical issues 11
2.3. Feedback by other routes 11
3. Other information 12
3.1. Monitoring and oversight 12
3.2. Quality assurance, ethical considerations and approvals 12
3.3. Data Storage, Archiving and Data Sharing 12
3.4. Project email mailing list 12
3.5. Transparency 13
Appendices: Dissemination plan 14

1. Background and Purpose

This document should be read alongside the PeRSEVERE 'Principles and Explanation' document, which goes into more detail about the project background and rationale. The main output from the PeRSEVERE project will be a set of broad, high-level principles to guide how the ethical right to withdraw informed consent in research should be put into practice.

The PeRSEVERE collaborative group has agreed to conduct a consultation exercise before finalising and releasing the principles (see **Figure 1**, below, for how this fits within the whole project plan). The objectives of this consultation are as follows:

1. Gather further feedback from relevant stakeholders on the draft principles, to inform further development.
2. Engage with the same relevant stakeholders with a view to raising awareness of the issues PeRSEVERE covers, starting conversations on the topic and ultimately influencing how the right to withdraw is implemented in practice (thereby improving how this is done from trial participants' point of view, ensuring trial integrity is protected as far as possible, and improving transparency of trial reporting).

This document describes the planned consultation exercise and how it will be disseminated to those we would like to reach.

Figure 1: overview of PeRSEVERE stakeholder consultation

Draft PeRSEVERE principles (complete)

↓

Consult stakeholders (at least 3 months)

↓

Revise principles and explanatory text based on feedback (approximately 2 months)

↓

Finalise principles and publicise (aim: end of 2021)

2. Consultation

2.1. Overview

Feedback will be sought directly through a survey, and also via less formal means (for example in response to presentations on this project). Each of these is described in more detail, below.

2.2. Feedback by survey

2.2.1. Survey eligibility

The survey will be publicly available for anyone to respond, though some principles may be more relevant to people who design and run trials.

Early in the survey, respondents will be asked if they primarily consider themselves a professional (i.e. running, designing, overseeing or funding trials, or with some other professional role in trials) or a patient, carer or member of the public (no professional role in trials, with or without experience of patient and public involvement [PPI]). This will be a key distinction, and we will only be able to conclude that our principles are broadly acceptable if we get agreement across the respondent groups.

We will make clear that we expect people to respond as individuals, rather than on behalf of groups, though we will encourage respondents to share the survey with colleagues and members of groups they are in. Also, as engagement with relevant groups and organisations could increase the impact of PeRSEVERE, we will encourage respondents to get in touch separately (via the PeRSEVERE team email address) if a group or organisation they are part of might be interested and able to help take the work forward following the survey.

Members of the PeRSEVERE steering group will not be eligible to complete the survey, and this will be made clear to the group when the survey is disseminated. They may still give feedback but it will be shared directly within the steering group rather than collected via the survey (and handled as per section 2.3, below). This group will still be encouraged to share the survey link with colleagues.

Members of the wider PeRSEVERE collaboration, i.e. those who have been involved in previous discussions about the work and/or who have reviewed previous iterations of the principles but have not led on principle development, may complete the survey. A question early in the survey will ask respondents if they have had any prior involvement with the project, to enable an assessment of how far beyond this initial group the survey has reached.

2.2.2. Recruitment and consent

We would like as many survey responses as possible (within limits of what we can feasibly analyse; see section 2.2.5 below for our minimum target sample size), and if possible from a wide range of respondents, e.g. patients and professionals, industry and academia, people from different countries

and people with different personal characteristics (e.g. ethnicity, gender, age). We will therefore disseminate the survey link widely, and encourage others to share onward. More details of the survey dissemination plan are given in the **Appendix,** at the end of this document. We will in particular aim to reach groups who we would like to influence with the final principles, namely:

- Patients, carers and potential/actual clinical trial participants;
- Charities and other third sector organisations with involvement or interest in research;
- Those designing and conducting clinical trials, including academic and industry researchers, clinicians and research practitioners (e.g. research nurses);
- Those approving and overseeing research, including ethics committee members, regulators and the Health Research Authority;
- Research funders.

In line with HRA guidance on proportionate consent,[1] consent to participate in the survey will be presumed to have been given when people choose to complete it. This approach will be made clear in the survey introductory text. The survey will not ask anyone to provide identifiable personal or confidential data. We will set up a mailing list for individuals to stay in touch with the project (see section 3.4, below), but this will be completely separate to the survey and personal details will not be linked to survey responses.

2.2.3. Survey design and data collection

The survey will contain the following elements:

- Introductory page(s):
  - Outlining the background and aims of the work;
  - Giving advance notice of the topics covered by the principles so people know what to expect (as a way to encourage completion of all questions);
  - Giving an indication of how many questions there are and therefore how much time it might take to complete the survey (although this could vary significantly depending on how many principles respondents comment on);
  - Introducing the format of the principles, and the questions about the principles;
  - Explaining that completion of any of the survey will be taken as implied consent to complete it;
  - Confirming that the survey will ask for no identifiable personal or confidential data and that none should be entered (a link to the University of Leeds research transparency policy[2] will nonetheless be provided);
  - Confirming that, as responses cannot be linked back to individuals, there is no way to remove responses once they have been entered;

[1] https://www.hra.nhs.uk/media/documents/applying-proportionate-approach-process-seeking-consent_R3gbJKn.pdf
[2] https://dataprotection.leeds.ac.uk/research-participant-privacy-notice/

    - Confirming that there will be no financial incentive or other payment available for completing the survey;
    - Giving information about how the survey feedback will be handled, and reiterating that the principles are not final and can be changed in response to the feedback received.
    - Giving project contact details in case of any questions.

- Initial questions:
    - Whether the respondent considers themselves primarily a research professional or primarily a non-professional (with or without PPI experience);
    - Whether or not they have taken part in research before as a participant, and if so whether they stopped any elements of participation early;
    - Whether or not they have heard of PeRSEVERE prior to hearing about the survey (to assess how far we have reached beyond the initial collaborative group).

- Overview of all principles:
    - A brief overview of the key messages from all 16 draft principles, to give respondents a chance to understand them as a collection before giving feedback on individual principles.
    - A single question asking for initial feedback on the list of key messages (to ensure some feedback is gained from all respondents, even if they skip the remaining sections without giving additional responses).

- Introductory page for each group of principles:
    - This will explain what the group of principles is about, and give participants an opportunity to skip them if they want to.

- Questions about each principle:
    - Each principle will be presented on screen, with an expandable-collapsible section giving more explanation about what each principle means and why it is suggested to be necessary. There will also be an expandable-collapsible glossary section to define any specific terminology in plainer terms, where needed.
    - Respondents will be asked to rate their agreement with several statements about each principle on a 5-point Likert scale (Strongly Agree, Agree, Not sure, Disagree, Strongly Disagree). The statements will be:
        - "This principle is clear and easy to understand" (to assess clarity);
        - "I can see how this principle could be put into practice" (to assess feasibility);
        - "I agree with what this principle says" (to assess acceptability);
        - "The principle already reflects my experience of running and/or taking part in research" (to assess novelty, i.e. how much each principle is or is not already established in practice).
    - A free text question will ask respondents if they have any feedback on the principle.

- Additional questions about terminology when talking about the right to withdraw consent in research.

- Demographics questions, with a message that completion of these is optional but encouraged:
  - Role/involvement in trials (categories)
  - Which clinical areas are relevant (if any), based on research experience and/or lived experience (categories)
  - Types of research worked on or participated in (categories)
  - If professional role, amount of time working in trials (Categories: 0-5 years, 6-10 years, 11-20 years, 21+ years)
  - Experience of working on research in academia, industry or both
  - If PPI contributor, amount of time doing that (Categories: 0-5 years, 6-10 years, 11-20 years, 21+ years)
  - Country based in
  - Age range
  - Gender (self-identified)
  - Ethnicity (self-identified)
  - Whether or not English is a first language

- A space to suggest any additional principles or topics for principles, if respondents feel something is missing from the existing set.
- A space to enter any other relevant comments.
- A thank you message and invitation to join the project mailing list (with up-front confirmation that personal details will not be linked to survey responses; see section 3.4 below for more on the mailing list).

The survey will be designed in Jisc Online Surveys.[3] The survey content has been reviewed by the steering group before finalisation, including the patient and site staff members. We have also piloted the survey with relevant individuals before finalising it.

There are no technical controls to prevent individuals from completing the survey more than once (even from the same computer), but we do not suspect anyone would have strong motivation to do this.

The survey will initially be open for 3 months, but this may be extended if the PeRSEVERE steering group agrees we need more feedback (in general or from specific groups of respondents).

2.2.4. Handling survey feedback

2.2.4.1. Data management, data cleaning and handling missing data

[3] https://www.onlinesurveys.ac.uk/

During analysis, we will highlight any inconsistent or illogical results (e.g. free text comments that seem to conflict with responses to quantitative questions) and discuss and handle these appropriately.

We will include missing data in our descriptive statistics. We will not impute any data, except potentially in the situation where a qualitative response has been left missing but the related free text response unambiguously indicates an answer (e.g. “I completely agree with this”). Any such imputation will be clearly documented and reported in the final project outputs.

All survey feedback will be downloaded prior to analysis and stored in a secure file location within the Clinical Trials Research Unit, University of Leeds. The PeRSEVERE project lead will scan the free-text responses for any identifiable information added (despite the instructions not to do this), and remove it immediately if any is found.

2.2.4.2. Quantitative data analysis

Descriptive analyses during and after the project will include:

- Responses to the per-principle quantitative questions, including median response (coded 1-5, with 5 being ‘strongly agree’), interquartile range and graphical summaries.
- Per-principle quantitative responses (as above) within the primarily professional and primarily non-professional respondent groups; we may also perform exploratory analysis of agreement across the additional demographic groups.
- Median responses (coded 1-5, with 5 being ‘strongly agree’) to the optional questions about suggested terminology, including within the primarily professional and primarily non-professional respondent groups.
- Summary of responses to demographics questions.

The analysis populations will be:

- For the demographics questions at the start and end of the survey and the ‘initial response’ question: all survey respondents who enter and save any data into the survey;
- For the per-principle quantitative questions: all survey respondents who said they wanted to give per-principle feedback for the relevant group of principles.

2.2.4.3. Qualitative data analysis

Each item of qualitative feedback for each principle will be reviewed and coded by a member of the PeRSEVERE steering group. If more than one individual performs this work, we will compare coding results across the coders after each coder has reviewed their first 10 survey responses. This will be in order to check agreement, and address any differences of understanding as required.

Each free text comment will be initially coded deductively using the framework of the quantitative questions that go with each principle, i.e. whether each comment is about clarity, feasibility, acceptability, novelty, something else or no comment (e.g. ‘No comment’ or adding nothing to the quantitative responses [‘I agree’]). Each free text comment will then also be coded inductively, i.e.

categorised based on what each comment says. Where possible, these granular categories will then be drawn together into broader inductive categories.

We will double-check the coding of 10% of the survey responses for each coder (or 100 responses if 10% is higher than 100). Re-coding and further checking will be carried out in responses to the initial double-check findings if, in the view of the checker(s), there are serious concerns about accuracy or consistency of coding.

For each principle, all free text comments will be summarised in terms of the deductively and inductively coded data, as above (e.g. total number of responses in each category).

The additional free-text questions at the end of the survey (about suggested terminology and about any other feedback the respondent wants to give) will be coded inductively, and will not be included in the double-checking of responses unless the other planned checking indicates possible problems with coding.

2.2.4.4. Monitoring and reporting

Quantitative data may be summarised periodically during the consultation period and presented to the steering group (as simple, descriptive analyses) for information and to guide any required action on further dissemination work, e.g. to increase the number of responses from certain groups.

All data will also be summarised for a peer-reviewed journal article once the project is complete. This would not identify or single-out any individual responses.

2.2.4.5. Informing further development of PeRSEVERE principles

Once the survey is closed to new responses, members of the PeRSEVERE steering group, including at least those coordinating development of each principle, will be presented with summarised survey feedback data for consideration. If feasible, members of the wider ‘writing groups’ who were involved in principle development will be invited to participate in this part of the process.

The participants in this activity will be asked to review the summarised survey responses using the points below as a guide (where median values are used to make decisions, this will always be reviewed alongside the frequency distribution to inform understanding of the responses):

- If the median responses to the questions about principle clarity and acceptability indicate agreement or strong agreement (i.e. a median score of at least 4), across both the primarily professional and primarily non-professional groups, then the current principle wording is unlikely to need substantial change.
- If the median score in either respondent group is less than 4 for either of these questions, the summarised qualitative data will be used to assess what improvements might be needed.
- If the median response from either of the respondent groups to the question about principle feasibility indicates lack of agreement (i.e. median less than 4), this is not enough to suggest a change in principle wording on its own (i.e. without corresponding concerns being raised about clarity or acceptability, as above), but it will inform how we disseminate the results of

our project. We will use the summarised qualitative data to understand the feasibility concerns, and include this information in our project outputs.

- The question about principle novelty will inform the interpretation and dissemination of our project. For example, if a given principle is agreed to be clear, feasible, acceptable *and* novel then we may need to highlight this as an area with more work still to do than a principle that is already well-established in practice. The answers to this question are unlikely to suggest a change to principle text.
- Qualitative feedback not fitting into any of these four areas will be reviewed separately and a suitable response agreed.
- We will be mindful of missing data in each case before reaching conclusions; i.e. if many survey respondents have missed out responses to the quantitative questions.
- Although we may give more weight to comments that are repeatedly made by survey respondents, we will not discount valid points made by only a few (or individual) respondents.
- In general, we will adopt a conservative approach to amending principles. Continually updating the principles in response to new feedback could result in a lot of changes and would give too much weight to new feedback, when the most up-to-date principles already reflect all the feedback given so far. When considering potential changes to the principles, the groups reviewing feedback will therefore be asked to consider:
    - Is the suggested change in the scope of PeRSEVERE (as defined in the 'Principles and Explanation' document)?
    - Might the change make the principle too long? Fundamental points will be included, but consideration will be given to whether it might be possible to reduce words elsewhere, or split the principle up.
    - Has the issue being raised already been discussed and a position already agreed?
    - Is it high-level enough? The PeRSEVERE principles are generally high-level, about *what* should happen rather than *how* it should happen. The 'how' should go with further implementation guidance rather than principle text.
    - Is a change actually being suggested by the commenter? In some cases they might be talking about a specific related issue, while not actually disagreeing with the fundamental principle message.

The feedback that is not principle-specific (i.e. the summarised responses to the additional free-text questions at the end of the survey) will be presented to the PeRSEVERE steering group for considerations in the same way as the principle-specific process described above.

A record of all discussion and agreed actions will be maintained. The PeRSEVERE project lead will be responsible for amending the 'Principles and Explanation' document as required following feedback review.

The feedback on the suggested terminology will also be presented to the steering group if time permits. The group will consider this feedback and agree suitable actions in response.

2.2.5. Sample size

As the survey will be used to gather feedback rather than to test a specific hypothesis, we will not perform a formal sample size calculation. However, we have set an arbitrary aim of 500 responses as a subjective measure of overall success of the survey. The PeRSEVERE steering group will also monitor accumulating response data to ensure there are (subjectively) adequate numbers of responses from different respondent groups (without setting specific pre-survey targets for these). If the response rate is unexpectedly high, the steering group will consider closing the survey earlier than planned to keep analysis manageable.

2.2.6. Ethical issues

We do not anticipate that this survey will raise any significant ethical issues. Respondents will be asked general questions about their views on a broad topic in clinical research (namely, how the right to withdraw informed consent should be put into practice). Although we cannot exclude some respondents being upset if particular principles remind them of something in their past, no sensitive information is collected in these questions. Some of the demographics questions could be considered sensitive, but we have consciously aimed to word them generally and non-intrusively. Survey completion is completely voluntary and optional, with no negative consequences of skipping questions or abandoning the survey partway through.

No identifiable personal or confidential data will be requested in the survey (the mailing list mentioned in section 3.4 will be entirely separate), and the introductory text will remind people not to enter anything identifiable in any free text fields. Should something identifiable be added by a respondent, it will be deleted by the PeRSEVERE project lead before the data is shared with anyone else.

2.3. Feedback by other routes

During the consultation period, we will plan to discuss the PeRSEVERE project with relevant groups and individuals, e.g. at webinars or similar events. This may provide an additional route for gathering feedback. Any suggestions gathered in this way will be documented and processed as ‘other’ feedback, as mentioned in 2.2.4.5, above. Where appropriate, individuals giving feedback will be asked to complete the demographics questions from the survey.

3. Other information

3.1. Monitoring and oversight

During the consultation period, the PeRSEVERE steering group, consisting of both professional members and patients, will regularly monitor response rates overall and within demographic and stakeholder groups, to check if any different actions might be needed in terms of survey dissemination. The PeRSEVERE steering group will continue to provide general oversight of the project and guidance on any issues arising.

3.2. Quality assurance, ethical considerations and approvals

We will obtain University of Leeds ethical approval prior to survey dissemination (or written confirmation from the relevant ethics committee that approval is not required).

Based on advice from the Health Research Authority (HRA), we will not seek HRA approval prior to commencing this work as it is not needed. This is because we will not recruit NHS staff by virtue of their role as NHS staff, but rather by general dissemination routes that may include NHS staff. We will make clear in the survey introduction that we do not have HRA approval (because it is not needed) and that if this might pose a problem for any potential participants then they should not complete the survey.

3.3. Data Storage, Archiving and Data Sharing

Survey responses will be collected within Online Surveys at University of Leeds. They will be downloaded and stored in a secure location at the Clinical Trials Research Unit (CTRU), University of Leeds. Data shared with other members of the steering group for review and/or analysis will not contain any information that could identify any survey respondents, and will be sent securely.

In line with University of Leeds data retention schedule[4], survey data will be retained for at least 5 years after publication of the main planned peer-reviewed paper about this work.

Data collected as part of this work will be available for further research on reasonable request, once any planned peer-reviewed publications are released. Data will be shared according to a controlled access approach, in line with CTRU policies. Data will only be shared in such a way that means there is no chance any individuals could be identified.

3.4. Project email mailing list

We intend to set up an email mailing list for anyone who would like to stay in touch with project progress, including about any project outputs and publications. This will be set up and maintained

[4] https://dataprotection.leeds.ac.uk/data-protection-and-personal-data/#how-long-can-I-keep-personal-data

separately to the consultation survey in a separate mailing list service run by Jisc. Sign-up will be completely optional and all potential subscribers will be provided with clear information about how their personal data will be processed if they sign up. They will be able to unsubscribe at any time using information provided to them by email.

The mailing list and its data will be retained only for as long as it is in use to send messages about the project. After this time, it will be securely destroyed.

3.5. Transparency

This project is not eligible for registration on clinical trials registries such as ISRCTN or clinicaltrials.gov. However, for transparency, we will make a copy of this consultation plan available on the UKCRC Registered Clinical Trials Unit Network website before opening the survey to responses.

Appendices: Dissemination plan

Overall plan

The survey will be disseminated via various routes, aiming to reach a wide range of people, both professionals and non-professionals, in the UK and beyond. Respondents will be encouraged to share onward.

Routes

- Direct dissemination:
    - Members of the PeRSEVERE steering group, patient group and wider collaborative group will be asked to share details of the survey with their contacts, if they are willing to do so. We will consider directly contacting influential individuals in the clinical research area, if appropriate. As per section 2.2.1 above, members of the steering group (including the patient group) will be informed that they may not complete the survey, but members of the wider collaborative group may.
    - Details of the survey will be distributed directly in messages and newsletters from the UK CRC Registered CTU Network.
    - We will contact some individuals and groups directly, to find out if they or colleagues may be able to complete the survey. These may include:
        - Academics and methodologists with relevant prior publications
        - Association of Medical Research Charities
        - Clinical Trials Transformation Initiative
        - Contacts in working in the commercial trial sector
        - Good Clinical Trials Collaborative
        - Health Data Research UK
        - Health Research Authority
        - Individuals who have asked to be kept updated about the project
        - Information Commissioner's Office (UK)
        - Inserm
        - Medical journal editors
        - Medical Research Council Regulatory Support Centre
        - Medicines and Healthcare products Regulatory Agency
        - NHS Digital
        - People in Research
        - Research funders
        - UK Research Ethics Committee members

- Indirect dissemination:
    - We will contact owners of other distribution routes (e.g. newsletters and mailing lists) and ask if they are willing to distribute the survey link to their subscribers/members (as well completing the survey themselves).

- These may include:
    - For mainly a patient- or public-facing audience:
        - Age UK
        - Alzheimer's Society
        - Arthritis Research UK
        - Asthma UK
        - British Heart Foundation
        - British Lymphology Society
        - British Skin Foundation
        - Cancer Research UK
        - Centre for BME Health (Leicester)
        - Centre for Evidence Based Dermatology
        - Cochrane (including the Consumer Network)
        - Cystic Fibrosis Trust
        - Diabetes UK
        - Healthwatch organisations
        - Heart Research UK
        - Independent Cancer Patient Voice
        - JDRF (diabetes charity)
        - Leeds Involving People
        - Lymphoedema Support Network
        - Lymphoma Action
        - National Eczema Society
        - Marie Curie
        - Mind
        - MND Scotland
        - MS Society
        - NCRI Consumer Forum
        - NIHR patient and public involvement contributors and professionals
        - North West Coast Research Community
        - Parkinson's UK
        - Stroke Association
        - Testicular cancer charities or campaigns (e.g. Orchid, It's on the ball, It's in the bag)
        - UK Community Advisory Board
        - Understanding Patient Data
        - Use My Data
        - Yorkshire Cancer Community

    - For mainly a research professional audience:
        - Academic collaborative groups (e.g. the MiDIA missing data group linked to London School of Hygiene and Tropical Medicine)
        - Association of Lead Research Nurses (https://auklrn.org/)
        - Association of the British Pharmaceutical Industry (IBPI)

- Health Research Board Trials Methodology Research Network (Ireland)
- NIHR mailing lists to reach research staff in the NHS (clinicians, research nurses and others)
- PSI (Statisticians in the Pharmaceutical Industry)
- Royal Statistical Society
- Trial Managers' Network
- Trials Methodology Research Partnership (UK)

- Twitter:
    - We will disseminate the survey link via Twitter, using template messages given below. Primarily this will be via the UK CRC Registered CTU Network Twitter account (https://twitter.com/ukctunetwork) but we will also retweet from other accounts owned by or linked to members of the PeRSEVERE steering group, and ask others to retweet on our behalf.

We may also give webinars or lead other engagement activities to publicise this project during the consultation. Feedback gathered in this way will be addressed as per section 2.3, above.

Template messages

The following are general templates for introducing the survey; they may be amended in small ways to suit the specific audience.

1. **Email invitation**

Dear…

We would like to invite you to take part in a consultation on how the right to withdraw informed consent in research should be put into practice. Please feel free to ignore this invitation if it is not relevant to you.

This consultation is part of the PeRSEVERE project (PRincipleS for handling end-of-participation EVEnts in clinical trials REsearch). PeRSEVERE is a collaborative project within the UK Clinical Research Network Registered Clinical Trials Unit Network (https://www.ukcrc-ctu.org.uk/default.aspx).

The project aims to help ensure that the right to withdraw informed consent is put into practice in a way that protects both individual participants' rights and the quality of research. We have seen that common misunderstandings about the right to withdrawal sometimes lead to:

- Research participants stopping elements of their participation that they have not said they wanted to stop;

- Research results being less reliable than they might be because less data is available for that analysis than might be;
- Research results being reported less clearly than they might be.

Through a collaboration involving a large group of research professionals and patients, we have developed broad, high-level principles to help achieve the aims set out above. We would now like your views on these principles. We would like your feedback whether or not you agree with our suggestions, and whether or not you agree there is a problem to resolve.

You can give as much or as little time as you like to this survey. If you choose to complete all the questions, it might take you at least 20-30 minutes, but it is possible to move through it much more quickly if you do not have that much time.

You can find the survey link and read more detail before agreeing to take part at: **[weblink].** The survey has been approved by **[details of ethics committee].**

Best wishes,

2. **Twitter messages**

General message

- The @ukctunetwork has been discussing how best to put the right to withdraw consent into practice to protect patients' rights and research integrity as part of the Persevere project. Now we want your input. Go to **[link]** to find out more.

Principle-specific messages

- Participation in #clinicaltrials can stop, but also change or reduce, if participants want that – according to #Persevere principle O1. Do you agree? Give your view at **[link]**
- Everyone running or taking part in #clinicaltrials should be aware that the more of the trial's data we collect, the better the results will be – according to #Persevere principle O2. Do you agree? Give your view at **[link]**
- Researchers losing contact with #clinicaltrial participants is not the same as participants saying they want to stop taking part – according to #Persevere principle O3. Do you agree? Give your view at **[link]**
- Data collection in #clinicaltrials should carry on until participants say they want it to stop – according to #Persevere principle O4. Do you agree? Give your view at **[link]**
- Data collected in a #clinicaltrial should be kept after a participant stops taking part, and should be made available for further, legitimate research – according to #Persevere principle O5. Do you agree? Give your view at **[link]**

- #clinicaltrials should be designed to cope with participants wanting to stop taking part early, and should have mechanisms to continue data collection wherever possible – according to #Persevere principle D1. Do you agree? Give your view at **[link]**
- #clinicaltrial protocols should contain all necessary information about what to do when trial participants want to stop or change their participation or when researchers lose contact with participants – according to #Persevere principle D2. Do you agree? Give your view at **[link]**
- Statistical planning for #clinicaltrials should include adequate prospective methods for participants wanting to stop or change their participation – according to #Persevere principle D3. Do you agree? Give your view at **[link]**
- #clinicaltrial participants should get adequate, clear and balanced information about stopping participation early, when they are considering taking part – according to #Persevere principle D4. Do you agree? Give your view at **[link]**
- #clinicaltrial participants should get clear information about what will happen if they and the researchers lose contact during the trial – according to #Persevere principle D5. Do you agree? Give your view at **[link]**
- #clinicaltrial researchers should encourage participants to discuss how they are finding trial participation, and should be prepared to discuss changes to participation if these might help – according to #Persevere principle D6. Do you agree? Give your view at **[link]**
- Everyone running #clinicaltrials should be adequately trained and supported to deal with participation changes in a way that protects participants' rights and trial integrity, wherever possible – according to #Persevere principle D7. Do you agree? Give your view at **[link]**
- Researchers running #clinicaltrials should collect informative data on participation changes to support trial management, monitoring and analysis – according to #Persevere principle M1. Do you agree? Give your view at **[link]**
- Researchers running #clinicaltrials should regularly review information on participation changes in case they need to take any action to protect the trial or its participants – according to #Persevere principle M2. Do you agree? Give your view at **[link]**
- Participation changes in #clinicaltrials should be transparently reported – according to #Persevere principle R1. Do you agree? Give your view at **[link]**
- #clinicaltrial results should be made available to each trial's participants, including those who stopped participating early – according to #Persevere principle R2. Do you agree? Give your view at **[link]**

Timing and reminders

- Invitation messages to different groups and routes may be staggered over a period of weeks or even months in order to reach a wider audience, and also for practical reasons (i.e. it will not be easy to send all messages at the same time).
- We will not systematically send reminder messages, but we may consider sending them to particular individuals or groups if it is appropriate.
