# Supplementary material for: Standardising management of consent withdrawal and other clinical trial participation changes: The UKCRC Registered Clinical Trials Unit Network’s PeRSEVERE project
Source: Clin Trials. 2025 Jul 4;22(5):578–96. doi: 10.1177/17407745251344524 (PMC12476473; doi:10.1177/17407745251344524)
Supplement: sj-pdf-7-ctj-10.1177_17407745251344524 – Supplemental material for Standardising management of consent withdrawal and other clinical trial participation changes: The UKCRC Registered Clinical Trials Unit Network’s PeRSEVERE project [file sj-pdf-7-ctj-10.1177_17407745251344524.pdf]

# PeRSEVERE consultation survey text v1.0 11-Mar-2021

## What is this project about? (Intro 1/4)

Thank you for visiting this consultation.

All participants in research studies have the right to withdraw their informed consent and stop participating whenever they like.

The PeRSEVERE project (principles for handling end of participation events in clinical trials research) is about how that right is put into practice. We argue it should be done in such a way that we can do the best by individual study participants, by each individual study and by research in general.

We have developed some principles to guide practice, and we now want feedback on these from a range of people - including patients, study participants, and people who run or oversee research studies.

There are 16 principles to look at, if you want to. The amount of time this survey takes you to complete will depend on how much feedback you want to give. It might take at least 20-30 minutes, especially if you choose to complete all the sections. However, you can also move through the survey more quickly if you do not have that much time.

**If you are interested to contribute to this, but might find it difficult to complete an online survey for any reason, please contact persevere@leeds.ac.uk to find out about other options.**

## More background information on PeRSEVERE (Intro 2/4)

This page gives some more brief background to PeRSEVERE, with links to more information if you need it. You do not need to read through this background information if you don't want to, but it may help you understand the thinking behind our draft principles.

**What is the scope of PeRSEVERE?**

PeRSEVERE does not include all types of research, or all issues to do with research participation changing.

| | |
|---|---|
| [Text appearing on clicking the link for more information] | 1. PeRSEVERE is about research where participants give informed consent and then have some ongoing involvement for a period of time. This includes most clinical trials, but excludes, for example, research where people give consent for their biological samples to be used but then have no other ongoing involvement.<br><br>2. PeRSEVERE is mainly about changes in research participation that affect how much data is available for analysis at the end of research. So research participants deciding to stop attending clinical trial hospital visits, for example, is relevant. But participants deciding to stop taking treatment, but agreeing to carry on attending all hospital visits is mostly not relevant (because this should not affect how much data is available for analysis).<br><br>3. PeRSEVERE is mostly not about improving 'retention' in clinical trials and other research, for example taking appropriate actions to make participants less likely to want to stop taking part. This is a very important area, but is outside of our scope. Instead, our starting point is that we accept that some research participants may want to stop or change their participation over time, and we think researchers should be as well-prepared for this as possible.<br><br>4. Sometimes research participants might need to change or stop their participation due to changes in their ability to make decisions for themselves (usually this is called their 'capacity to consent'). There are specific laws and guidance about what should happen in those situations, and they are therefore generally outside of our scope. |

**How have the principles been developed?**

The draft PeRSEVERE principles have been developed through extensive discussion and debate by a broad group of research professionals and patients in the UK.

| | |
|---|---|
| [Text appearing on clicking the link for more information] | Our draft principles have been developed through extensive discussion and debate within our collaborative group, which was formed through the UK Clinical Research Collaboration Registered Clinical Trials Unit Network (https://www.ukcrc-ctu.org.uk/default.aspx) and which includes patients, statisticians, methodologists, trial and data managers and specialists in quality assurance and research regulation.<br><br>In developing our principles, we have been guided by the high-level principles of ethical research conduct (including good clinical practice), our knowledge and understanding of existing clinical research regulations and guidance, and our collective experience of designing, running, analysing, reporting on and participating in clinical trials.<br><br>In general, we suggest it is best to be proactive in ensuring the above aims are achieved. This means researchers should design trials and prepare to run them in the right way, rather than only reacting to problems as they occur. We have aimed to keep this in mind as we have developed the principles.<br><br>We acknowledge that some of our principles are already reasonably well-established in clinical research (at least in the UK). However, we consider it important to present them all as a complete, coherent set. We are not aware of any previous attempts to comprehensively define, as we have done here, how the ethical right of trial participants to withdraw their consent should be put into practice. |

**A note on terminology and language**

In our experience, there can be confusion and ambiguity around terms commonly used to describe stopping trial participation, such as ‘withdrawn’, ‘lost to follow-up’, ‘drop-out’ or ‘off-trial’.

In general in this survey, we aim to describe participation ending or changing as plainly as we can, using language with explicit meaning which, as a result, can mean the same thing to everyone.

You can give feedback on our suggested terminology at the end in the survey, if you would like to.

## How this survey will work (Intro 3/4)

### What do you want me to do?

We would like your views on our proposed principles. Although lots of people, including researchers and patients, have been involved in developing the principles so far, we want to make sure they are right. We would be really grateful for your feedback - each individual's responses are valuable to this work.

Please note that we would like you to respond as an individual, not on behalf of any groups or organisations. If a group or organisation you are in might be interested to help with the progression of this project, please feel free to get in touch at persevere@leeds.ac.uk.

### What will the questions look like?

| | |
|---|---|
| [Text appearing on clicking the link for more information] | On the following pages, we will present each principle, with some more explanation if you want it, and give you a chance to give your views.<br><br>For each principle, we will ask you about how clear it is, how easily you can see the principle being put into practice, how acceptable the principle is to you, and how much it already reflects your experience of research. There will also be a space to give feedback, including to explain your answers if you want to. All questions are optional and you can skip any of them without giving an answer if you want to or if you have run out of time.<br><br>There are 16 principles in total, but you can skip individual principles, or groups of them, if you don't want to give feedback on those. The principles are in four groups:<br><br>- Overarching principles (about the overall approaches to take when thinking about the right to withdraw informed consent)<br>- Trial design and participant information<br>- Data management and monitoring<br>- End of trial reporting and results dissemination<br><br>At the end of the survey, we will also ask a few general questions about you so that we have an idea of who has given feedback. It is important to us that a variety of people give feedback, both in terms of who they are and what they do. You do not have to complete these, but it will be very helpful to us if you do.<br><br>We do not expect any of the questions to ask you for sensitive information about you or your life, but if you feel uncertain or upset about any of the questions then of course please feel free to stop completing the survey, and your results will not be saved. |

**Can I save the survey and come back to finish it later?**

| | |
|---|---|
| [Text appearing on clicking the link for more information] | Yes, you can save the survey and return to it later, though we would encourage you to do it in one go if you possibly can.<br><br>If you do want to save and return, there is a link at the bottom of each page ("Finish later"). If you click this, you will be able to receive a link by email to return to the survey later. |

**What will happen to my feedback?**

| | |
|---|---|
| [Text appearing on clicking the link for more information] | The PeRSEVERE steering group, made up of researcher and patient members, will review all feedback. Where we agree a change needs to be made to the principles in response to your feedback, we will make it, though we can't guarantee that all feedback will lead to a change.<br><br>We intend to publish the results of this work in a peer-reviewed scientific journal. It will not be possible to identify you or any other survey respondents from this publication |

## What you need to know before you complete the survey (Intro 4/4)

### What else do I need to know before completing the survey?

If you decide to complete this survey, we will take this to mean you have consented to take part, based on the information given here.

We will not ask you for any information about you that could identify you, and please do not enter any. If you do enter anything identifiable, we will remove it before doing anything with the survey data.

There is a chance to join a project mailing list at the end of the survey, but this will be entirely separate from the survey, and we will not link your details with the answers you give. Your survey responses will be stored securely at the Clinical Trials Research Unit at the University of Leeds. For more information about how the University of Leeds protects research data, please go to https://dataprotection.leeds.ac.uk/wp-content/uploads/sites/48/2019/02/Research-Privacy-Notice.pdf.

This survey has obtained ethical approval from **[details]**. Based on Health Research Authority (HRA) guidance, we have not obtained HRA approval. If you feel this is a problem for any reason, please do not complete the survey in work time, or share it with colleagues.

### Can I ask you to delete my survey responses after I've completed the survey?

| | |
|---|---|
| [Text appearing on clicking the link for more information] | As there won't be any way to identify which responses are yours, it won't be possible to delete your survey responses after you've submitted them. |

### How long will you keep the survey responses and will you share them with anyone else?

| | |
|---|---|
| [Text appearing on clicking the link for more information] | We will keep the survey responses for at least 5 years after publication of the results of this project. After this, once the data is no longer useful for research purposes, we will securely destroy it.<br><br>We will only share data with people outside of the PeRSEVERE project team for valid additional research projects, and only in such a way that there is no way they could identify anyone from the survey responses. |

### Will I get any reimbursement or financial incentive for completing this survey?

| | |
|---|---|
| [Text appearing on clicking the | There is no money available for the time you give completing this survey. If this is a problem for you, then please do not complete the survey. |

| | |
|---|---|
| link for more information] | |

**What will happen to the results of this project?**

| | |
|---|---|
| [Text appearing on clicking the link for more information] | We intend to publish the results of this work in a peer-reviewed scientific journal. This won't identify any individual survey participants. If you want to find out the results of the project, you can sign up to the mailing list at the end of this survey. We will also share the results with relevant organisations and groups, so you might find out that way as well. |

**Can I share this survey with others?**

| | |
|---|---|
| [Text appearing on clicking the link for more information] | Yes! Please feel free to share it with anyone else who might be interested to help. |

If you have any questions about this survey or this project, please get in touch at persevere@leeds.ac.uk.

**By completing and submitting responses to this survey you are consenting to take part. Please confirm you understand this by answering this question.**

- **I consent to take part in this survey [required question]**

## What is your relationship to research?

**Before we begin, please could you let us know which of the following applies best to you? (Choose one)**

- I am a research professional / I am involved in research as part of my job
- I am a patient, carer or member of the public and I am involved in research through patient and public involvement (PPI) work
- I am a patient, carer or member of the public and I have no involvement in research in any capacity except as a potential/actual research participant
- Unsure / hard to say

**Have you ever taken part in research as a participant?**

- Yes
- No
- Not sure

**If you have been a research participant, did you stop/have you stopped taking part in any elements of the research early?**

(For example, did you stop taking study treatment, or stop attending study-specific hospital visits, before it was/they were due to stop? This could be because you decided to do this, or because someone else advised you to do it.)

- Yes
- No
- Not sure
- Not applicable

**Had you heard of the PeRSEVERE project before you heard about this survey?**

- Yes, and I have already been involved in developing the PeRSEVERE principles
- Yes, I had heard of it but I have not been involved in any way before now
- No

## Overview of all principles

Before you go to the individual principles, it will be useful to see an overview of all of them, so you can see what is included.

Below is a list of the **key messages** from each principle. The full principle text usually goes into a little more detail about what we mean, but these key messages are the main things to take from our project.

Group 1, overarching principles:

- Everyone running or taking part in studies should be aware that participants may choose to change, reduce or stop their participation after they agree to join the study.
- Everyone running or taking part in studies should be aware that the more of a study's planned data that is collected, the better.
- Losing contact with a participant should not be considered the same as a participant saying that they want to stop study participation.
- Data collection should continue until a study participant explicitly tells researchers that they want it to stop.
- Study data collected in accordance with the approved study protocol up to the point a study participant stops providing data should be used in the study analysis.

Group 2, study development and participant information:

- Studies should be designed and resourced to allow data collection to continue wherever possible, particularly for study outcome data.
- Study protocols should include clear instructions on how participation status changes should be managed.
- Study protocols and statistical analysis plans should include considerations for the impact of participation changes on planned statistical analysis.
- Before participants agree to take part they should receive clear and balanced information about what will happen if they want to stop participating.
- Participants should be informed before they consent to join a study what will happen if contact is lost during the study.
- Throughout the study, researchers should regularly check if participants are happy taking part and should be prepared to discuss changes to participation if required.
- Everyone involved in running studies should be trained and supported to manage participation changes for the good of both the participants and the study.

Group 3, data management and monitoring:

- Data about study participation changes should be recorded in a standardised way and include information to usefully inform study management, analysis and reporting.
- Researchers responsible for running and overseeing a study should, at appropriately regular intervals, review data about participation changes in the study.

Group 4, study reporting:

- End of study reporting of participation status should be consistent within a study, showing any changes in level of participation, preferably by randomised group.
- All study participants should be offered the opportunity to receive the study results when they are available and thanked for their contribution, regardless of any changes to their study participation.

We would like to know your initial reaction to these key messages, if possible. The questions in the rest of the survey ask for more detailed feedback about each principle.

We would encourage you to give the more detailed feedback if you have time, especially if you disagree with any of the points, or if any of them were not totally clear. However, it is possible to move through the rest of the survey quickly if you do not have much time. You can also easily go to the questions about a particular topic, if you are interested to comment on it.

We are grateful for any feedback you give, no matter how much detail you give.

**What is your initial response to the key messages listed above? Choose the statement that fits best.**

- I totally agree with all of the key messages
- I mostly agree with the key messages
- I agree with some of the key messages
- I do not agree with the key messages at all
- Not sure

## Overarching principles

The next few principles are in the 'overarching' group. They cover overall issues of how we think of the 'right to withdraw' in research, and how it should be handled.

**There are five principles in this group:**

- **O1: participation can stop, reduce or change**
- **O2: the more data, the better**
- **O3: losing contact**
- **O4: continuing data collection**
- **O5: retaining data**

These principles cover some important points. We are therefore keen to get feedback on these from everyone who completes the survey, and we would encourage you to look at these questions if you can.

However, if you do not want to give feedback on these principles, you can skip to the next group, which is about how studies should be set up and what information should be given to study participants.

**Would you like to read these principles in more detail and have the chance to give feedback?**

- Yes, I would like to read them
- No, I will skip to the next section

**[Format of this introductory page repeated for each of the other 3 sections: study design and participant information; data management and monitoring; end of study reporting and results dissemination]**

## Principles O1-O5: overarching principles

### Principle O1: Participation can stop, reduce or change

Everyone running or taking part in studies should be aware that participants may choose to change, reduce or stop their participation after they agree to join the study.

All language and communication about any changes to participation should be clear about exactly what has changed, and what has not.

| | |
|---|---|
| [Text appearing on clicking the link for more information] | In laws and other rules about research with human participants, 'withdrawal' of informed consent is described in all-or-nothing terms – participants have given consent to participate, or they have withdrawn their consent and are not taking part in any aspects of the study anymore.<br><br>In reality, things are often more complex than that. Study participants might choose to completely stop some aspects of study participation, for example taking study treatment, filling in study questionnaires, or attending study-specific hospital visits. Rather than stopping participation entirely, they might instead want to reduce participation, for example taking part less often. If possible within the study, participants might also change how they take part, for example they might take part in study activities at the same times, but in a different way (for example, via telephone calls with their research nurse instead of going into a clinic for a visit). Unless a participation change needs to happen in order to protect a study participation (usually in relation to whether or not they continue taking a study treatment), all changes in participation should be decided by the participants themselves.<br><br>It is important that everyone running and taking part in studies is aware of this complexity. We also need to use language that reflects this. Just describing study participants as 'withdrawn' or 'off-study' is unlikely to be useful in many cases, because we won't know exactly what participants' wishes are, or exactly what they want to stop.<br><br>See also:<br><br>See the principles about study development D1 (protecting study integrity by design), D2 (protocol content) and D3 (statistical planning) for more on preparing for participation changes during the study design stage.<br><br>The issue of clear language and communication mentioned above affects many aspects of how studies are designed, run and reported. As |

| | |
|---|---|
| | well as study protocols (see principle D2), this is important in communication between researchers and study participants (see principles D4, D5 and D6), training of researchers running studies (see principle D7), data collection (principle M1), monitoring study progress (principle M2) and study reporting (R1). |

**Glossary**

| | |
|---|---|
| [Text appearing on clicking the link for more information] | Study protocol: this is the document (or set of documents) that describes why a study is needed, what it aims to achieve and how it should be run. |

**Please indicate how much you agree with the following statements in relation to this principle.**

Please don't select more than 1 answer(s) per row.

| | Strongly agree | Agree | Not sure | Disagree | Strongly disagree |
|---|---|---|---|---|---|
| This principle is clear and easy to understand | ☐ | ☐ | ☐ | ☐ | ☐ |
| I can see how this principle could be put into practice | ☐ | ☐ | ☐ | ☐ | ☐ |
| I agree with what this principle says | ☐ | ☐ | ☐ | ☐ | ☐ |
| This principle already reflects my experience of running and/or taking part in research | ☐ | ☐ | ☐ | ☐ | ☐ |

**Do you have any feedback on this principle?**

[Space for free-text response]

**[Format of text and questions for this principle is repeated for each of the other 15 principles; see list above for the key messages of each principle, and see the live survey link and/or the 'principles and explanation' document for full text that will be presented to survey participants in the format shown above]**

## Optional: how should we talk about study participation changing?

In our experience, there can be confusion and ambiguity around terms commonly used to describe stopping or changing study participation, such as ‘withdrawn’, ‘lost to follow-up’, ‘drop-out’ or ‘off-study’.

**How clear do you find currently used terminology about participation changes?**

For example: 'withdrawn', 'lost to follow-up', 'off-study', 'drop-out'

- Very clear
- Somewhat clear
- Not sure
- Somewhat unclear
- Very unclear

In general, we aim in our project to describe participation ending or changing as plainly as we can, using language with explicit meaning which, as a result, can mean the same thing to everyone.

Some suggestions for clearer terminology are outlined below. We suggest these, or similarly clear terminology, could be used in many situations when designing, running, analysing and reporting studies, including in study protocols, study reports, and in communications between researchers and study participants.

This level of detail may not be necessary in all situations. For example, by the time of a study’s final analysis, it may be useful to simply summarise which participants we have outcome data for and which we do not. However, use of the suggested terminology below does not prevent this sort of summarisation taking place when it is needed.

**Click 'more info', below, to see our suggestions for clearer terminology. If you do not have time or are not interested in feeding back about this, please just click through to the next page.**

| | |
|---|---|
| [Text appearing on clicking the link for more information] | **For aspects of study participation requiring ongoing commitment (such as receiving study treatment, attendance at study-specific clinic visits, completing questionnaires):**<br><br>When a participant decides to completely stop an aspect of participation before it was supposed to finish, we suggest using the phrase **"stopped … early"**. For example "stopped study-specific visits early".<br><br>In some cases, it might be useful to distinguish between participation changes following a participant’s decision and changes (particularly to |

| | |
|---|---|
| | medical treatment or procedures) guided by clinical decisions of a participant's doctors. For example, if a participant's doctor decided it was in their best interests to stop receiving a study treatment, they could be described as having "stopped treatment early based on a clinical decision".<br><br>When a participant decides to reduce their level of commitment, without totally having stopped, we suggest using the term **"reduced…"**. For example, "reduced frequency of study visits". This can also be used to describe changes in receipt of study treatment or intervention, but study protocols often make specific provisions around dose delays or modifications (if this is the case, the protocol's terminology should be used).<br><br>When some alternative, specific arrangement has been made with a participant regarding their participation, we suggest this should be explicitly stated, for example "stopped study visits early; telephone follow-up only". Alternatively, the general term **"changed…"** can be used, for example "follow-up changed from study visits to telephone follow-up only".<br><br>When a participant has stopped an aspect of participation at the time that the protocol specified it was supposed to stop, we suggest using **"completed…"**. For example, "completed study visits".<br><br>In some studies, there may not be a set period of time when participants get treatment/intervention. For example, participants may continue receiving treatment until it does not seem to be working any more. In these cases, the idea of stopping treatment 'early' might not be quite right, but nor might it be right to say they have 'completed' treatment. In these cases, we suggest the phase **"(permanently) stopped treatment because [of]…"**. For example, "stopped treatment because it was no longer beneficial", or "stopped treatment because of side effects".<br><br>**For aspects of study participation that do not require ongoing, active commitment** (e.g. researchers getting additional data for the study from routine healthcare data providers like NHS digital, or biological samples being stored for future research projects) we will say **"no longer agrees to…"**. For example, "the participant no longer agrees to have their biological sample stored for future research". |

| | |
|---|---|
| | **Loss of contact:** we expect study protocols to specify how to manage loss of contact with participants (which means participants stopping study participation without having expressed any explicit wish about stopping). This should include a set process to follow, and criteria for judging when to stop trying to contact someone (or stop for the time being). At this point, we suggest describing the person as having **"lost contact for now"**. This implies that it should be possible to try again at a later date to contact them, for example prior to the final study analysis. The protocol should specify when this later contact should be.<br><br>At the end of the study, there may be a group of participants who lost contact with the study without explicitly asking to stop participating and for whom no further information was ever obtained. We suggest these people could be described as having **"lost contact and never regained, with no further follow-up."**<br><br>If data about these participants is obtained through other sources (such as from routine healthcare data providers like NHS Digital, and always in line with participant consent), they might instead be categorised as having had **"lost contact and never regained, but with some indirect follow-up".**<br><br>**General terms:** we suggest **"change in participation status"** as a general term used to mean all instances where a participant makes a decision (or in some cases where a decision is made on their behalf) to end or reduce study participation.<br><br>The terms above are not mutually exclusive, as ending or changing participation can be complex and specific to an individual's situation. The terms can be combined as appropriate with "but" or "and" in order to convey exactly what has stopped, reduced or still continues in each case. For example, "the participant stopped intervention early and is on reduced study visits, but still agrees to their data being accessed through routine healthcare data sources". |

**Please indicate how much you agree with the following statements in relation to our suggestions, above.**

Please don't select more than 1 answer(s) per row.

| | Strongly agree | Agree | Not sure | Disagree | Strongly disagree |
|---|---|---|---|---|---|

| | | | | | |
|---|---|---|---|---|---|
| The suggestions are clear and easy to understand | ☐ | ☐ | ☐ | ☐ | ☐ |
| The suggestions are an improvement on the language people tend to use at the moment. | ☐ | ☐ | ☐ | ☐ | ☐ |

**Do you have any feedback on these suggestions?**

[Space for free-text response]

## More about you

You can skip this page if you like, but it would be very useful to us to know a little about you so that we can check that we have heard a range of views. The PeRSEVERE steering group will review this information to see if we need to make more effort to reach certain groups of people. We may also report a summary of this information (without any individuals being identified or singled out) in a peer-reviewed journal article.

**Which of these apply to you? (Tick all that apply)**

- Bioethicist / ethicist
- Charity or other third sector organisation (professional or volunteer)
- Clinician / medical doctor
- Epidemiologist
- Ethics committee member
- Information Systems / Information Technology expert
- Involved in day-to-day research management (e.g. trial manager)
- Involved in research data management (including data entry or data cleaning)
- Journal editor
- Leadership role (e.g. Director) within clinical trials unit or other research organisation
- Patient/carer/member of the public without a professional research role
- Patient and public involvement contributor
- Pharmacist
- Programmer / Systems Developer
- Regulator / involved in research oversight
- Research funder / involved in decisions about research funding
- Research methodologist
- Research nurse / practitioner
- Research quality assurance professional
- Statistician/analyst
- Employed by or involved in another organisation with a research interest
- None of the options above are relevant to me

**Which of these are relevant to you, either as a researcher or a patient? (Tick all that apply)**

- Alzheimer's disease or dementia
- Cancer
- Conditions affecting pregnancy or childbirth
- Conditions affecting the blood (e.g. anaemia, haemophilia)
- Conditions affecting the digestive system
- Conditions affecting the ears or eyes
- Conditions affecting the immune system
- Conditions affecting the kidneys or urogenital system (e.g. urinary tract infections)

- Conditions affecting the lungs or breathing (e.g. asthma)
- Conditions affecting the muscles or skeleton
- Conditions affecting the nervous system (e.g. epilepsy or Parkinson's disease or MS)
- Conditions affecting the skin
- Conditions people are born with (congenital conditions)
- Dentistry
- Diabetes
- Emergency healthcare
- Healthcare for children
- Heart or circulation problems (e.g. high blood pressure or stroke)
- Infectious diseases
- Mental health
- Other metabolic or endocrine disorders (e.g. thyroid problems)
- Primary care
- None of the above / none in particular / not sure

**Which of the following do you have experience of working with and/or participating in? (Tick all that apply)**

- Clinical trials of medicines (also known as 'CTIMPs')
- Cluster-randomised studies
- Complex intervention studies
- Diagnostics or screening studies (e.g. cancer screening)
- Disease management studies
- Early phase clinical trials (for example, 'first-in-man' studies or phase one studies)
- Epidemiology studies
- Health research in low or middle income countries
- Health services research
- Late phase clinical trials (phase II, III or IV studies)
- Medical device studies
- Medical imaging studies
- Public health research
- Questionnaire-based health research (e.g. surveys about health)
- Radiotherapy studies
- Social care research
- Surgery studies
- Vaccine studies
- None of the above / none in particular / not sure

**If you have or have had a professional role in research, how long have you had/did you have this for?**

- 0-5 years

- 6-10 years
- 11-20 years
- 21+ years
- Not applicable

**If you have or have had a professional role in research, what sector was/is this in?**

- Public sector / academia
- Commercial sector / pharmaceutical company
- Both public and commercial sector
- Other / hard to say
- Not applicable

**If you are a patient who contributes or has contributed to patient and public involvement, how long have you done/did you do this for?**

- 0-5 years
- 6-10 years
- 11-20 years
- 21+ years
- Not applicable

**Which country are you mainly based in?**

[List of countries]

**How old are you?**

- Younger than 30 years old
- 30-45 years old
- 46-65 years old
- 66+ years old

**How would you describe your gender?**

- Male
- Female
- Neither of the above categories
- Prefer not to say

**How would you describe your ethnicity?**

- Asian
- Black
- Mixed or multiple ethnicities
- White
- None of the above categories
- Prefer not to say

**Is English your first language?**

- Yes
- No
- Not sure / hard to say

## Any other comments?

**Have we missed anything? Are there any other important things to consider in preparing for and dealing with participants who stop trial participation early?**

[Space to give free-text answer]

**Do you have any further comments about this work or the issue of participation changes and withdrawal of consent in clinical trials? Please do not enter anything that might identify you or anyone else.**

[Space to give free-text answer]

## Final page

Thank you very much for giving feedback in this consultation.

If you would like to stay in touch with this project, please visit **[link]** to join our mailing list. This is completely optional, and if you choose to sign up, your personal details will not be linked up to the answers you have given just now.

If you have any further questions for the PeRSEVERE project team, suggestions for how we should take this work forward or ideas for how our principles should be put into practice, please get in touch at persevere@leeds.ac.uk.
