# Supplementary material for: Standardising management of consent withdrawal and other clinical trial participation changes: The UKCRC Registered Clinical Trials Unit Network’s PeRSEVERE project
Source: Clin Trials. 2025 Jul 4;22(5):578–96. doi: 10.1177/17407745251344524 (PMC12476473; doi:10.1177/17407745251344524)
Supplement: sj-pdf-8-ctj-10.1177_17407745251344524 – Supplemental material for Standardising management of consent withdrawal and other clinical trial participation changes: The UKCRC Registered Clinical Trials Unit Network’s PeRSEVERE project [file sj-pdf-8-ctj-10.1177_17407745251344524.pdf]

Below is an example scenario illustrating how the PeRSEVERE principles can be put into practice to help decide what to do in ongoing clinical trials, and to help prepare for (or even prevent) such scenarios. Further example scenarios are available at: https://perseverеprinciples.org/scenarios-persevere-in-practice/

A participant has previously decided to stop trial intervention and trial-related visits, but continues to see the same doctor as part of routine care. The doctor (who is the main investigator at the trial site) knows that the final scheduled questionnaire is particularly important for the trial's objectives.

The doctor has a discussion with the participant (instigated by the participant) at a routine healthcare visit about the overall trial progress. This leads to the participant subsequently deciding to complete the questionnaire during the visit.

Some on the trial team have suggested that the participant should formally reconsent to the trial by completing another consent form, before the questionnaire can be used in the trial. However, it seems this will be difficult to do at short notice as the participant lives quite far from the site.

- **Can the questionnaire data be used for the trial?**
    - It may not be right to assume the questionnaire data cannot be used – the participant might well have done it willingly, so discarding the data might not be fair to them.
    - Look at what the participant said before about how they wanted their participation to change. How does this new data provision fit with that?
    - Trial researchers need to be reassured that the participant was not pressured or coerced into completing the questionnaire. It might be relevant to consider whether there is a big risk of coercion in this context, or how high the 'stakes' are for the different people involved. Might it be possible for the participant to confirm their wishes to someone other than the trial doctor?
    - Questionnaire completion is not without risk for the participant, but is relatively low-risk compared to some other trial activities. It is often considered in-scope for 'proportionate approaches' to consent. Might we consider the participant completing this questionnaire to imply their consent?
    - Completing a new consent form will be burdensome for the participant, especially if it needs to be done in person. There is unlikely to be an ethically approved consent form just for this purpose. So it may not be feasible to do

this – is it fair to the participant to discard the information they provided, just because of this?
  - Excluding the data from the trial will have a negative impact on the trial results. Is that justified in this case?

- **How can the Persevere principles help prepare for this?**
  - **Principle O2:** the extent of participation change is the participant's choice. This means participants can change their mind about stopping participation, as long as it still helps meet the trial objectives.
  - **Principle O3:** decisions about participation should be informed by understanding of the value of collecting the trial data. This means a participant could make an informed, freely-given choice to provide one last questionnaire, with the knowledge that it could be helpful to the trial.
  - **Principle O7:** it is OK to keep a participant informed about the trial even after they've stopped taking part. The participant might just be interested to hear how the trial is going, even if they have no intention of making any more contributions to it.
  - **Principle D1:** plan to allow reduced participation (e.g. completion only of final questionnaire) if participants might be interested to keep making a contribution. The trial team in this case might have built the possibility into the protocol, so that if a participant is thinking of stopping participation, they could be offered the chance to stop all the questionnaires apart from the last one, if they want.
  - **Principle D5:** encourage dialogue about participation to help discuss this sort of case. This might make it easier to discuss and agree for the participant to complete fewer questionnaires, rather than stopping them all.
  - **Principle D6:** site staff should get training on how to handle these sorts of cases. Research staff are keen to do the right thing, and it can be challenging to serve the needs of individual participants and trials at the same time. Training might increase staff confidence in managing the initial participation change, and the situation where a participant wants to re-start making a contribution to the trial.
  - **Principle M1:** ensure good record keeping about participation changes. This includes a clear record of how the participant wants their participation to change at different times in the trial. It also includes keeping a record of the discussions (for example in the participant's medical notes), particularly at the time of that final questionnaire.
